# Liposomes with Low Levels of Grafted Poly(ethylene glycol) Remain Susceptible to Destabilization by Anti-Poly(ethylene glycol) Antibodies

**DOI:** 10.1021/acsnano.4c05409

**Published:** 2024-08-09

**Authors:** Bing-Mae Chen, Even Chen, Yi-Chen Lin, Trieu Thi My Tran, Keren Turjeman, Shih-Hung Yang, Tian-Lu Cheng, Yechezkel Barenholz, Steve R. Roffler

**Affiliations:** † Institute of Biomedical Sciences, Academia Sinica, Taipei 11529, Taiwan; ‡ Graduate Institute of Life Sciences, National Defense Medical Center, Taipei 11490, Taiwan; § Department of Biochemistry and Molecular Biology, Hebrew University-Hadassah Medical School, Jerusalem 91120, Israel; ∥ Graduate Institute of Medicine, College of Medicine, Kaohsiung Medical University, Kaohsiung 80708, Taiwan

**Keywords:** anti-PEG antibodies, PEGylated liposomal doxorubicin, irinotecan liposomes, destabilization, complement activation

## Abstract

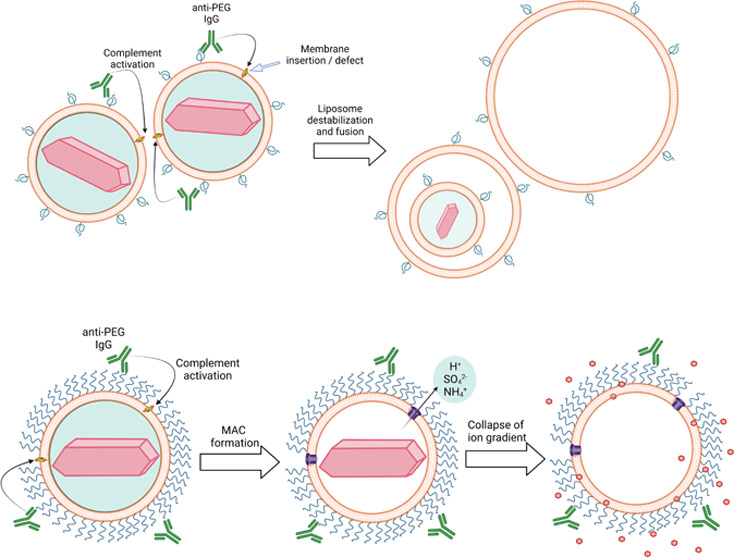

Binding of anti-PEG antibodies to poly(ethylene glycol) (PEG) on the surface of PEGylated liposomal doxorubicin (PLD) *in vitro* and in rats can activate complement and cause the rapid release of doxorubicin from the liposome interior. Here, we find that irinotecan liposomes (IL) and L-PLD, which have 16-fold lower levels of 1,2-distearoyl-*sn*-glycero-3-phosphoethanolamine (DSPE)-PEG_2000_ in their liposome membrane as compared to PLD, generate less complement activation but remain sensitive to destabilization and drug release by anti-PEG antibodies. Complement activation and liposome destabilization correlated with the theoretically estimated number of antibody molecules bound per liposome. Drug release from liposomes proceeded through the alternative complement pathway but was accelerated by the classical complement pathway. In contrast to PLD destabilization by anti-PEG immunoglobulin G (IgG), which proceeded by the insertion of membrane attack complexes in the lipid bilayer of otherwise intact PLD, anti-PEG IgG promoted the fusion of L-PLD, and IL to form unilamellar and oligo-vesicular liposomes. Anti-PEG immunoglobulin M (IgM) induced drug release from all liposomes (PLD, L-PLD, and IL) *via* the formation of unilamellar and oligo-vesicular liposomes. Anti-PEG IgG destabilized both PLD and L-PLD in rats, indicating that the reduction of PEG levels on liposomes is not an effective approach to prevent liposome destabilization by anti-PEG antibodies.

## Introduction

Poly(ethylene glycol) (PEG) is a biocompatible, nontoxic polymer that is attached to proteins, peptides, nucleic acids, nanoparticles, and liposomal carriers to increase their circulation time and therapeutic efficacy.^1,2^ However, the use of PEG in pharmaceuticals is under scrutiny because patients administered PEGylated drugs can produce antibodies that bind to PEG, and anti-PEG antibodies pre-exist in many healthy individuals.^3−7^ Anti-PEG antibodies have been linked to decreased clinical efficacy and increased side effects, thus becoming an important area of research.^8−14^

We recently reported that anti-PEG antibodies can activate complement and induce drug release from PEGylated liposomal doxorubicin (PLD).^15^ This was confirmed for PLD and extended to the release of mRNA from lipid nanoparticles.^16^ PLD consist of doxorubicin (DOX)-sulfate encapsulated as a nanorod crystal inside the aqueous phase of a liposomal carrier decorated with a dense (5.3 mol %) coat of PEG. We found that despite the presence of a protective layer of PEG, anti-PEG immunoglobulin G (IgG) antibodies activated serum complement to form membrane attack complexes in the liposome membrane, thereby disrupting the transmembrane ion gradient and causing doxorubicin to rapidly leak out of liposomes. Irinotecan liposomes (IL), which are used for the treatment of pancreatic cancer, is a liposomal formulation of irinotecan with a much lower density of PEG on its surface (0.3 mol % compared to 5.3 mol % on PLD).^17,18^

We hypothesized that the lower density of 1,2-distearoyl-*sn*-glycero-3-phosphoethanolamine (DSPE)-PEG present in IL may increase resistance to the effects of anti-PEG antibodies and offer a solution to destabilization by anti-PEG antibodies. In the present investigation, we studied whether anti-PEG antibodies can bind and induce drug release from IL despite the low density of DSPE-PEG in the IL membrane. We also created PLD with the same density of PEG as IL (0.3 mol %) to determine if any difference in the effects of anti-PEG antibodies is related to the density of membrane DSPE-PEG or other differences such as drug encapsulation methodology, drug organization inside the liposomes, drug properties, or liposome size. We report that IL and L-PLD remain susceptible to destabilization by anti-PEG antibodies, but the mechanism of liposome destabilization and drug release differs from PLD.

## Results and Discussion

### Anti-PEG Antibody Binding and Complement Activation to PLD and IL

**Figure 1:**
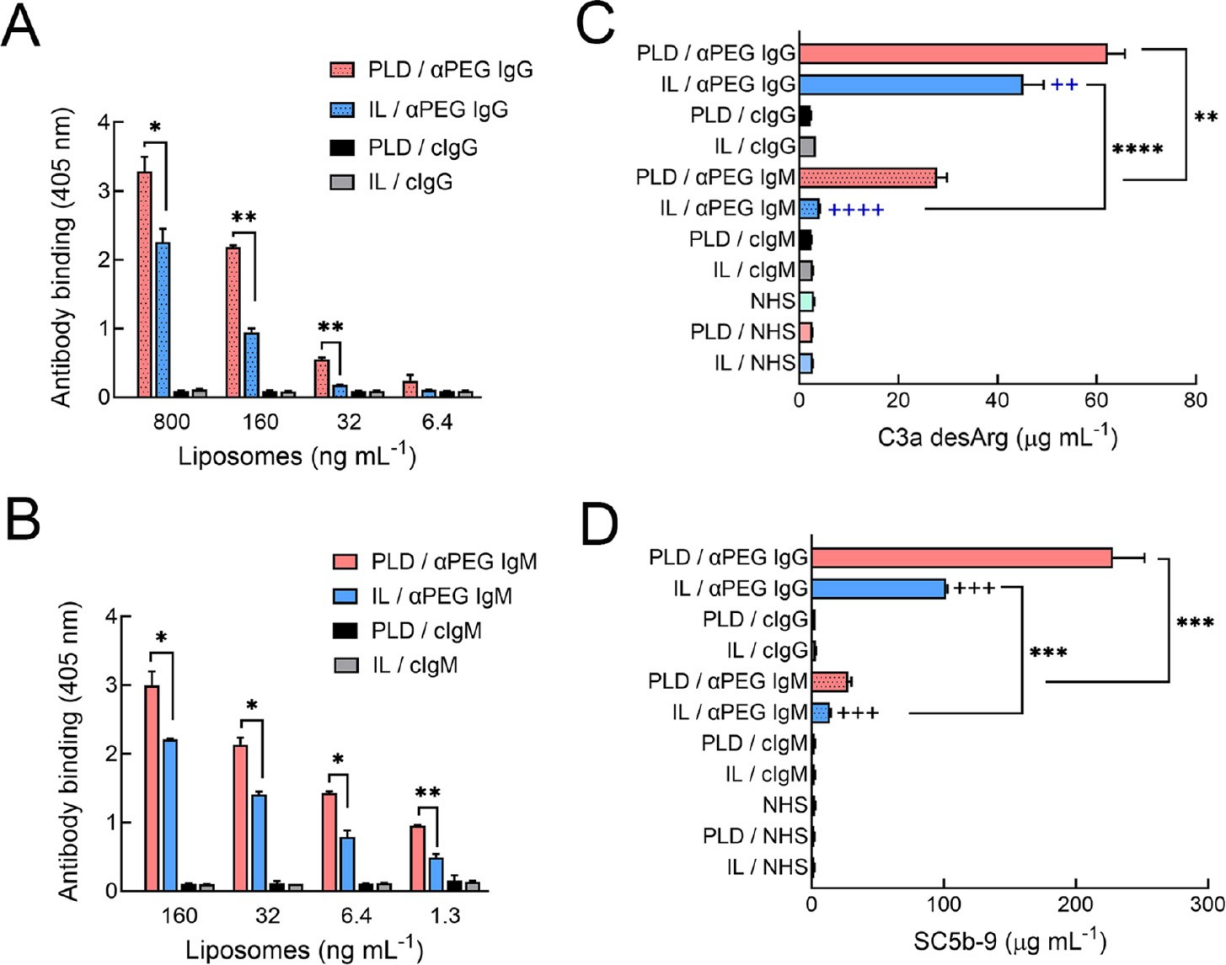
Humanized anti-PEG antibodies bind to PLD and IL and activate complement. Binding of 2.5 μg mL^–1^ human hu6.3 biotin-labeled anti-PEG IgG (A) or chimeric human cAGP4 anti-PEG IgM (B) antibodies to the indicated concentrations (based on lipids) of PLD or IL in duplicate are shown. cIgG and cIgM are isotype-matched negative control antibodies. The concentrations of C3a desArg (C) and SC5b-9 (D) after 150 μg mL^–1^ human anti-PEG antibodies were incubated in triplicate with 34.2 μg mL^–1^ PLD or IL in normal human serum (NHS) for 15 min. Bars show the mean values and standard deviations. Statistical significance between differences in mean values: *, *p* ≤ 0.05, **, *p* ≤ 0.005; ***, *p* ≤ 0.001; ****, *p* ≤ 0.0001. Significant differences between complement activation by PLD and IL are also indicated; ++, *p* ≤ 0.005; +++, *p* ≤ 0.001; ++++, *p* ≤ 0.0001.

We compared the binding of well-characterized humanized anti-PEG IgG (hu6.3) and chimeric human immunoglobulin M (IgM) (cAGP4) antibodies to PLD and IL.^4,15,26^ IL is a liposomal formulation of irinotecan which has about 16-fold less PEG grafted on the liposome surface as compared to PLD (one in 300 lipid molecules in IL compared to 1 in 20 molecules for PLD). Serial dilutions of PLD or IL were captured in microliter plates coated with a mouse antimethoxy PEG antibody before a fixed amount of biotin-labeled anti-PEG IgG or anti-PEG IgM was added to the wells. Biotin-labeled irrelevant human IgG and IgM antibodies were also assayed as binding controls. Antibody binding to liposomes was measured by adding horseradish peroxidase (HRP)-streptavidin to detect the biotin-labeled antibodies. Significantly more anti-PEG IgG (Figure 1A) and anti-PEG IgM (Figure 1B) bound to PLD as compared to IL, consistent with a higher density of PEG on PLD.

Complement activation by anti-PEG antibodies bound to PLD and IL was examined by incubating anti-PEG IgG or anti-PEG IgM with the liposomes in normal human serum at 37 °C and then measuring the complement reaction products C3a *desArg* and SC5b-9. Human serum was prescreened to ensure that it did not contain pre-existing anti-PEG antibodies. C3a *desArg* is a more stable form of C3a that rapidly forms in serum due to cleavage of the C-terminal arginine of C3 whereas SC5b-9 is a soluble form of the membrane attack complex.^27,28^ Anti-PEG IgG and IgM antibodies generated significantly higher concentrations of both C3a *desArg* (Figure 1C) and SC5b-9 (Figure 1D) from PLD as compared to IL, probably reflecting the higher antibody binding to PLD. Anti-PEG IgG induced significantly more complement activation than anti-PEG IgM for both PLD and IL (Figure 1C,D). These results indicate that anti-PEG IgG and anti-PEG IgM induce ∼25–75% (Figure 1A–D) less complement activation in the presence of IL as compared to PLD *in vitro*.

### Anti-PEG Antibodies Can Induce Drug Release from PLD and IL

**Figure 2:**
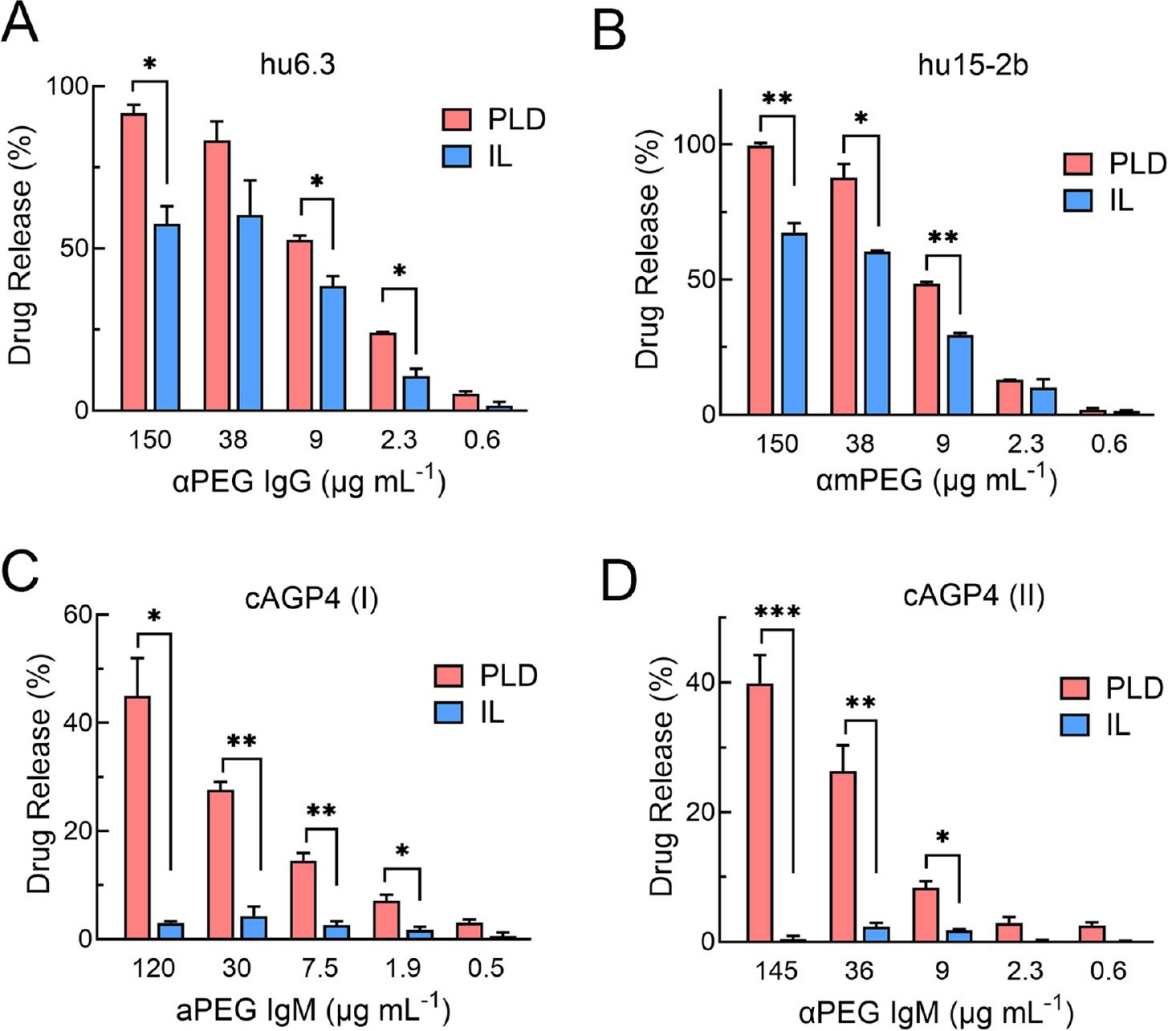
Comparison of drug release from PLD and IL. Drug release was measured from 34 μg mL^–1^ (based on lipid) PLD or IL incubated with the indicated concentrations of hu6.3 anti-PEG IgG (A), hu15-2b antimethoxy PEG (B) or two separate batches of cAGP4 anti-PEG IgM (C, D) for 30 min at 37 °C in human sera. Bars show standard deviation; *n* = 3. Statistical significance between differences in mean values: *, *p* ≤ 0.05, **, *p* ≤ 0.005; ***, *p* ≤ 0.001.

Liposome destabilization by anti-PEG antibodies was examined by incubating anti-PEG IgG or IgM with PLD or IL in normal human serum and measuring drug release after 30 min. Hu6.3 anti-PEG IgG destabilized both PLD and IL (∼90 and 50%, respectively), but significantly more doxorubicin was released from PLD as compared to irinotecan release from IL at the same concentration of hu6.3 anti-PEG IgG (Figure 2A). Incubation of liposomes with a humanized antibody (hu15-2b) with specificity for the terminal methoxy group of PEG also produced similarly strong drug release from both PLD and IL, with nearly complete drug release observed at 150 μg mL^–1^ antibody. By contrast, two independent preparations of cAGP4 anti-PEG IgM induced less total drug release from PLD (<50%) as compared to hu6.3 anti-PEG IgG and hu15-2b antimethoxy PEG IgG and very little (<10%) drug release from IL (Figure 2C,D). Nonbinding control IgG and IgM antibodies did not induce drug release from either PLD or IL (Figure S1), confirming the requirement for antibody binding to PEG for liposomal destabilization.

### Cryogenic Transmission Electron Microscopy (Cryo-TEM) Imaging of PLD and IL

**Figure 3:**
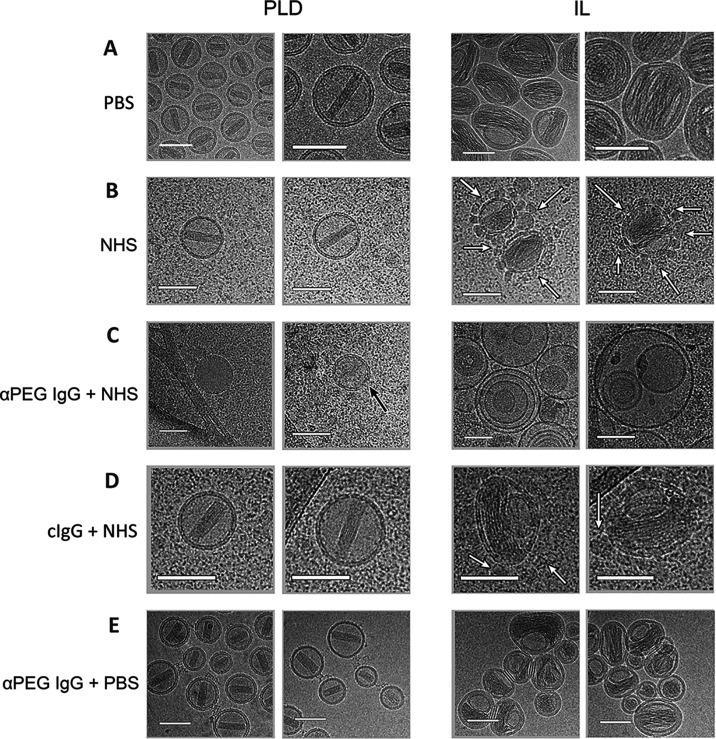
Cryo-TEM examining the effect of anti-PEG IgG on PLD and IL. PLD or IL were incubated for 15 min at 37 °C in (A) PBS, (B) normal human serum, (C) human serum containing 5 μg mL^–1^ anti-PEG IgG, (D) human serum containing 5 μg mL^–1^ control human IgG, or (E) PBS containing 5 μg mL^–1^ anti-PEG IgG. Black arrows indicate areas where the membrane has been damaged. White arrows indicate a “corona” on the outside of IL. All scale bars show 100 nm.

We used cryogenic transmission electron microscopy (cryo-TEM) to further examine the effects of anti-PEG antibodies on PLD and IL stability. In phosphate-buffered saline (PBS), PLD appear as circular or slightly elongated (elliptic) liposomes with a single nanorod-like crystal apparent in the intraliposome aqueous phase whereas IL appear as elongated, irregular liposomes with an extensive crystal-like structure present in the lumen (Figure 3A). Most PLD and IL liposomes have drug crystals in them. A clear corona could sometimes be seen surrounding the IL in human serum; the same phenomenon was less frequently observed for PLD (Figure 3B). When anti-PEG IgG was added to human serum, many empty PLD and IL were apparent, consistent with the release of drug from the liposomes. Empty PLD remained the same size but contained defects in the lipid membrane that likely represent pores formed by the insertion of the membrane attack complex in the lipid bilayer (Figures 3C left and S2). By contrast, many empty IL liposomes appeared to fuse together and form large oligolamellar and oligo-vesicular liposomes (Figures 3C, right and S3). A human IgG_1_ control antibody failed to induce drug release from PLD or IL in the presence of serum, indicating that PEG recognition by antibodies is important for drug release (Figure 3D). We previously demonstrated that PLD destabilization was prevented when complement activity was blocked.^15^ Likewise, heat or chemical deactivation of complement activity prevented IL destabilization by anti-PEG antibodies (Figure S4). The requirement for active complement for liposome destabilization is further shown by the lack of an effect of anti-PEG IgG on PLD or IL in PBS (Figure 3E).

**Figure 4:**
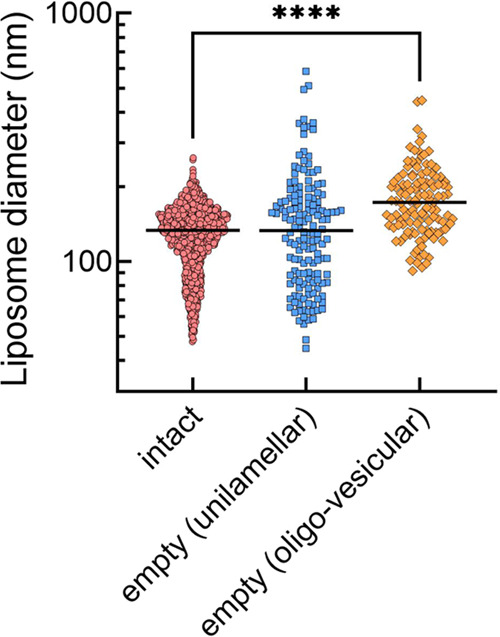
Sizes of the intact and empty IL. IL (68.5 μg mL^–1^ lipid) was incubated for 5 min at 37 °C with anti-PEG IgG (25 μg mL^–1^) in 50% human serum/50% GHBS^2+^ buffer. Cryo-TEM images were analyzed to determine the diameters of intact (*n* = 1686) IL, or empty IL that were unilamellar (*N* = 154) or oligo-vesicular (*n* = 121). Only the largest outside bilayer was measured in the multilamellar oligo-vesicular liposomes. Significant differences in median liposome diameters are indicated, ****p < 0.0001.

Examination of cryo-TEM images of IL incubated in human serum in the presence of hu6.3 anti-PEG IgG revealed that empty liposomes that formed after anti-PEG antibodies-induced drug release could be divided into those that remained unilamellar and those that became oligo-vesicular. Empty unilamellar IL retained the same median diameter as intact IL (Figure 4), but their size distribution span increased (Table 1). Empty oligo-vesicular IL, by contrast, displayed a size distribution span similar to that of intact IL, but the median diameter significantly increased as compared to intact IL (Figure 4). Interestingly, the calculation of liposome surface area based on mean diameters shows that the outside largest bilayer (104,000 nm^2^) of empty oligo-vesicular IL was about 2 times larger than the surface area of intact IL (52,700 nm^2^). Based on the final liposome size and the oliovesicular structure, it appears that anti-PEG antibodies can induce the fusion of liposomes.

**Table 1:** Size Distribution of IL Determined by Cryo-TEM after Treatment with Human Anti-PEG IgG in Human Serum^a^

	intact	empty due to drug release (unilamellar)	empty due to drug release (oligo-vesicular)
	Diameter (nm)		
*D*_10_	77.5	66.5	120
*D*_50_	133	132	172.5
*D*_90_	170	242	253
span (*D*_90_ – *D*_10_)/*D*_50_	0.70	1.33	0.77
number of liposomes	1686	154	121

a The diameter where 10% of the liposomes are smaller (*D*_10_), 50% of the liposomes are smaller (*D*_50_), and 90% of the liposomes are smaller (*D*_90_) are indicated. The outermost lipid bilayer of empty multilamellar oligo-vesicular liposomes was measured.

**Figure 5:**
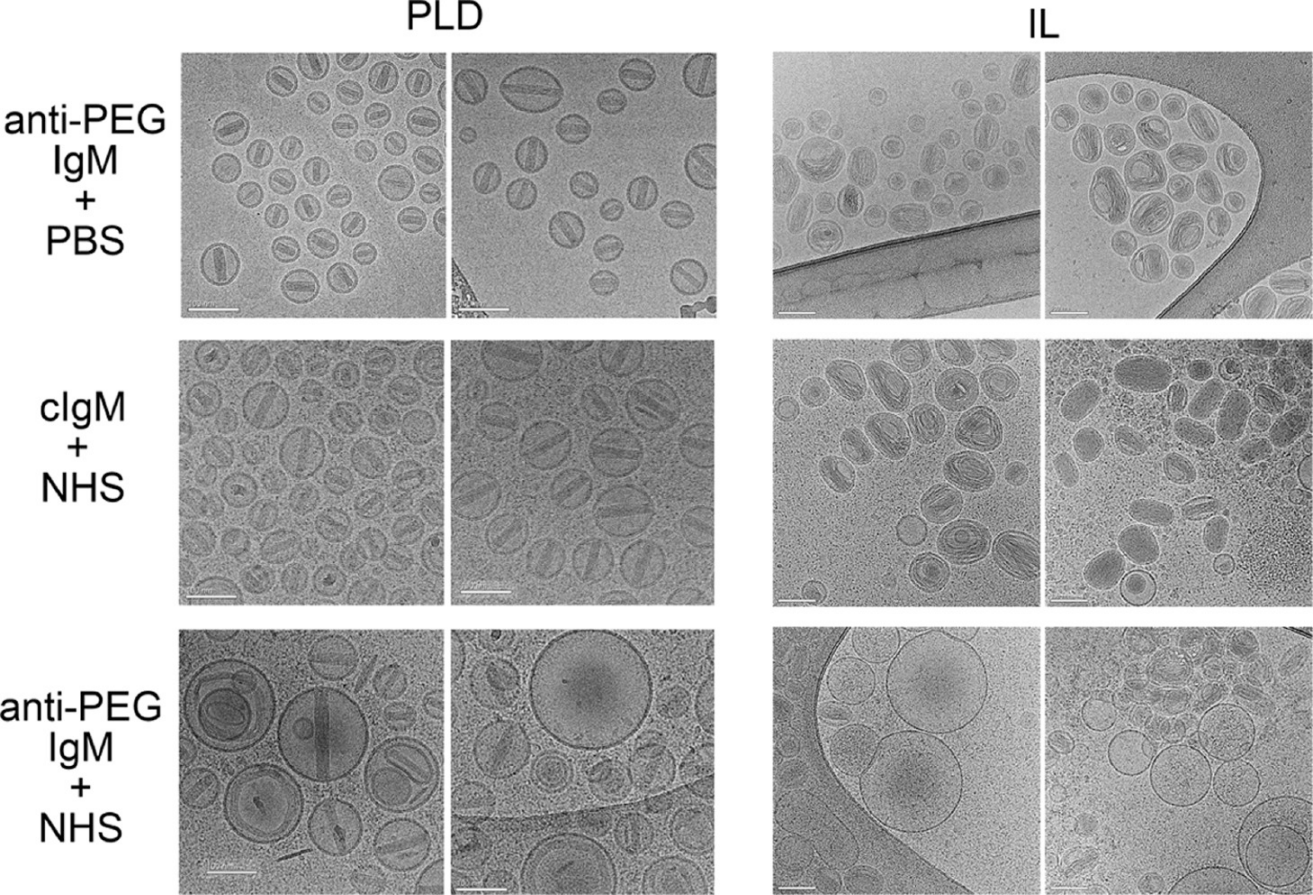
Cryo-TEM examining the effect of anti-PEG IgM on PLD and IL. PLD or IL were incubated for 15 min at 37 °C in PBS containing 5 μg mL^–1^ cAGP4 anti-PEG IgM (anti-PEG IgM + PBS), normal human serum containing 5 μg mL^–1^ control human IgM (cIgM + NHS), or normal human serum containing 5 μg mL^–1^ cAGP4 anti-PEG IgM (anti-PEG IgM + NHS). Scale bars = 100 nm.

We also examined the effect of anti-PEG IgM antibodies on PLD and IL by cryo-TEM. Surprisingly, large unilamellar and oligo-vesicular liposomes were observed when PLD was exposed to cAGP4 anti-PEG IgM in human serum (Figures 5 and S5). This is distinct from the effects of anti-PEG IgG, in which large unilamellar and oligo-vesicular liposomes were rarely observed. This may indicate that IgM can overcome the PEG barrier and cause direct interactions between liposomes. Similar to the effects of anti-PEG IgG on IL, anti-PEG IgM also caused IL to form large unilamellar and oligo-vesicular liposomes in the presence of human serum (Figures 5 and S6). PLD and IL were not destabilized in normal human serum when control IgM was added or when anti-PEG IgM was added in PBS, consistent with the requirement for complement activation for liposome destabilization by anti-PEG IgM.

### Differences between PLD and IL Reflect Differences in Surface PEG Density

**Figure 6:**
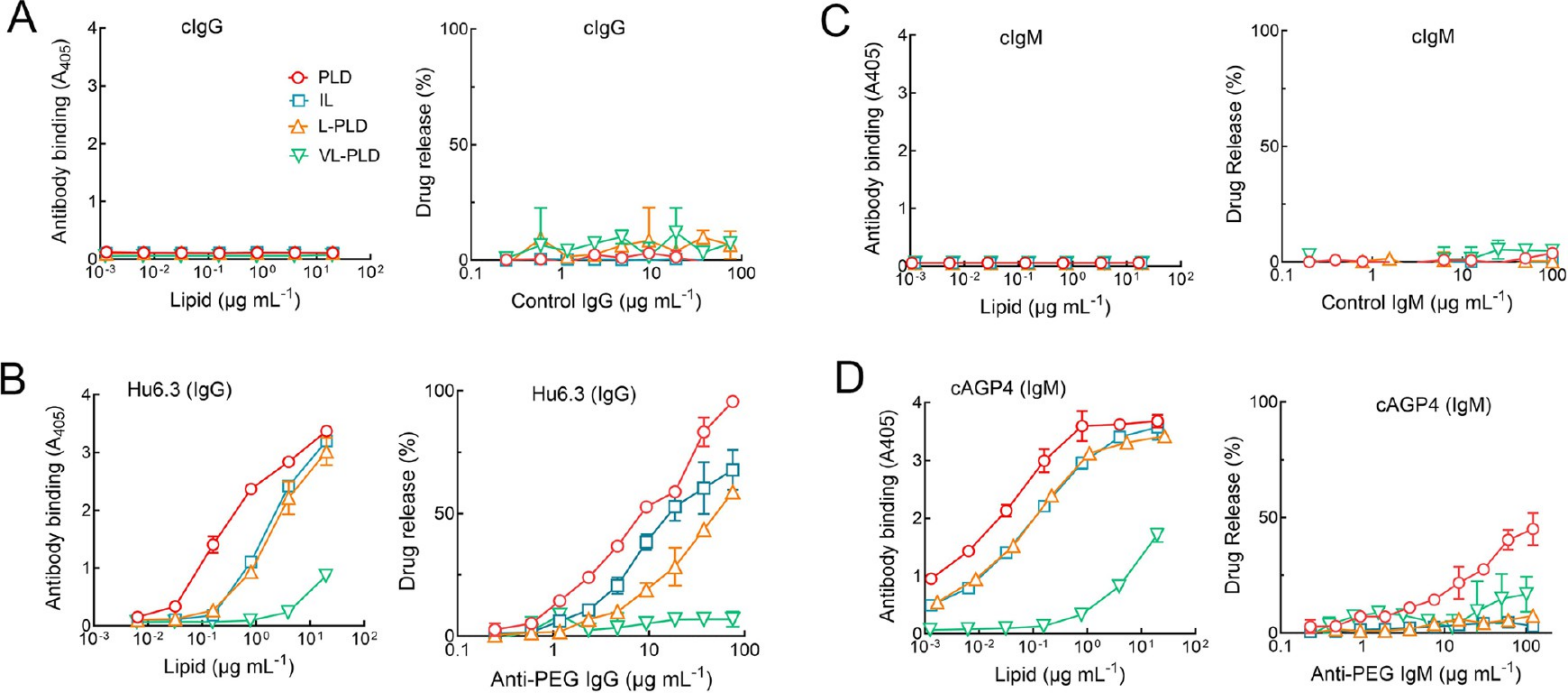
Anti-PEG antibody binding and drug release. Antibody binding (left panel) and drug release (right panel) were measured for the indicated liposomes incubated with (A) control IgG, (B) antihuman IgG, (C) control IgM, or (D) anti-PEG IgM. Antibody binding to liposomes was measured by capturing serial dilutions of liposomes (concentrations show total lipids) in microtiter plates and then incubated with 2.5 μg mL^–1^ biotin-labeled antibodies followed by streptavidin-HRP. Drug release was measured from 68.5 μg mL^–1^ (based on lipid) IL, PLD, L-PLD, or VL-PLD after incubation in human sera for 30 min at 37 °C in the presence of the indicated concentrations of human antibodies. Bars, SD; *n* = 2–3.

Besides different amounts of DSPE-PEG incorporated in PLD and IL, these formulations also differ in the properties of the loaded drug, the number of drug molecules per liposome, the internal loading buffer, liposome size distribution, and subtle variations in lipid composition. To further understand if differences in drug release primarily reflect the difference in PEG surface density, we produced PLD in which the level of DSPE-PEG was reduced from 5.3 mol % in PLD to 0.3 mol % (L-PLD) or 0.01 mol % (VL-PLD). The L-PLD has the same PEG surface density as IL. These liposomes were loaded with doxorubicin to the same level as found in PLD. Human control IgG did not bind to any of the liposomes (Figure 6A, left) and therefore did not induce consistent release of the internal drug cargoes from liposomes in the presence of normal human serum (Figure 6A, right), showing that antibody binding to liposomes is needed for complement activation and liposome destabilization. About 5-fold less hu6.3 anti-PEG IgG bound to IL and L-PLD and about 100-fold less anti-PEG IgG bound to VL-PLD as compared to PLD (Figure 6B, left panel). Anti-PEG IgG induced the release of more than 50% of doxorubicin from PLD, IL, and L-PLD but only low levels of doxorubicin (<10%) were released from VL-PLD at all anti-PEG IgG concentrations examined (Figure 6B, right panel). Drug release approximately mirrored antibody binding to the liposomes in the rank order PLD > IL > L-PLD ≫ VL-PLD. As expected, control IgM did not bind or induce drug release from any of the liposomal formulations (Figure 6C). The binding of cAGP4 anti-PEG IgM also followed the rank order PLD > IL and L-PLD ≫ VL-PLD (Figure 6D, left panel). Anti-PEG IgM induced moderate drug release from PLD (∼50% release at 100 μg mL^–1^) but very little drug release from the other liposomes (Figure 6D, right panel). These results indicate that differences in *in vitro* drug release from liposomes are well explained by differences in PEG coating density.

### Comparison of Anti-PEG Antibody-Mediated Complement Activation by PLD, IL, and L-PLD

**Figure 7:**
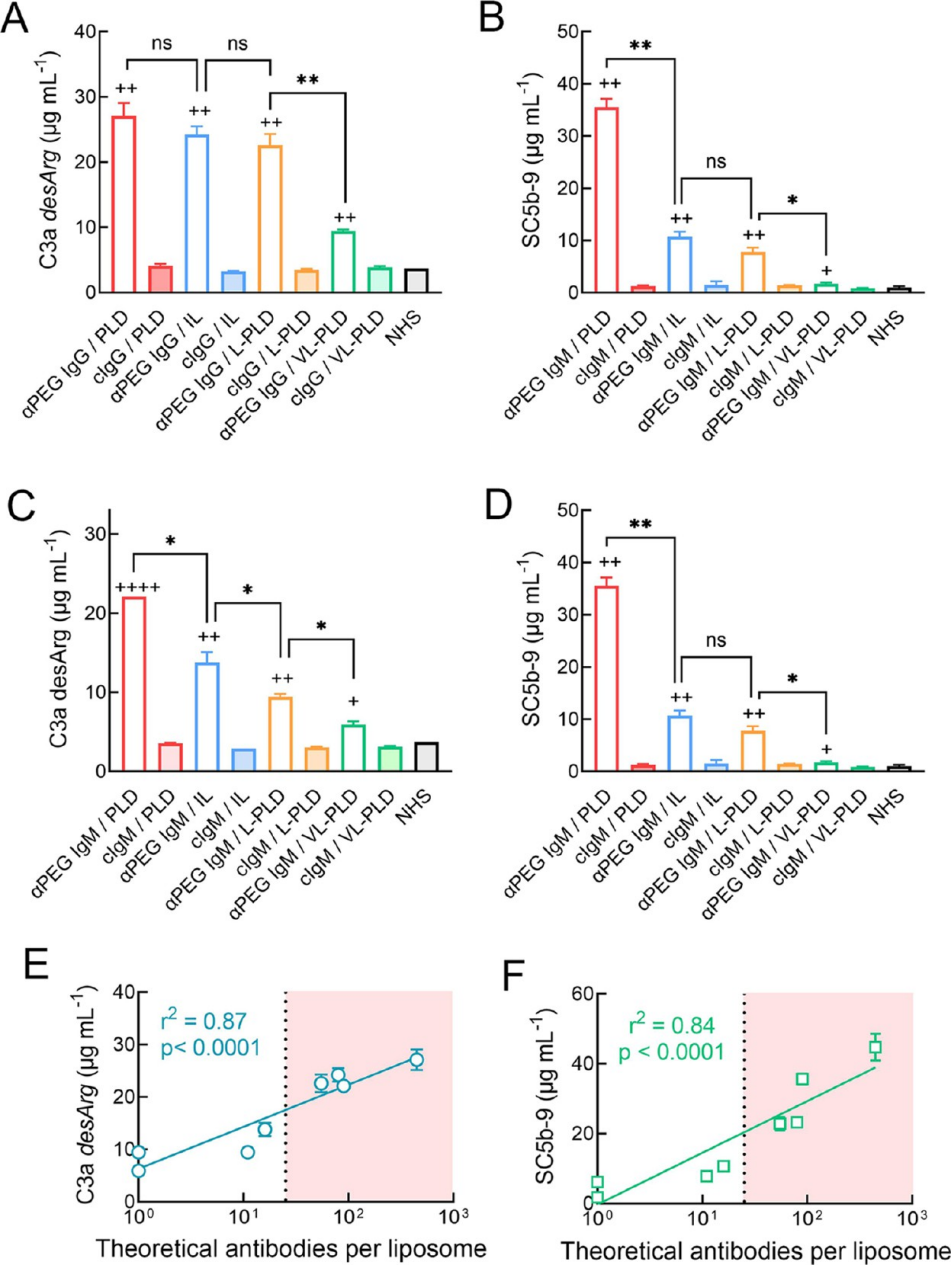
Liposome PEG mol % affects complement activation by anti-PEG antibodies. Human anti-PEG or control antibodies (75 μg mL^–1^) were incubated with 68.5 μg mL^–1^ PLD, IL, L-PLD, or VL-PLD in normal human serum for 15 min and then the concentrations of C3a desArg (A, C) and SC5b-9 (B, D) were measured by ELISA. Columns show mean values of triplicate determinations. Bars, SD. Statistical significance between anti-PEG and control antibodies: #, *p* ≤ 0.05; ##, *p* ≤ 0.005; ###, *p* ≤ 0.001; ####, *p* ≤ 0.0001. Significant differences between different liposomes treated with anti-PEG antibodies are also indicated: ns, not significant; *, *p* ≤ 0.05; **, *p* ≤ 0.005. Correlation between concentrations of C3a *desArg* (E) and SC5b-9 (F) and the theoretically estimated number of anti-PEG IgG and IgM antibodies bound on each liposome after incubation of human anti-PEG antibodies with 68.5 μg mL^–1^ PLD, IL, L-PLD, or VL-PLD in normal human serum. The line shows the least regression line for complement products *versus* log_10_ of the theoretically estimated number of bound antibodies per liposome. The shaded region indicates the region in which very strong drug release from liposomes was observed.

Both anti-PEG IgG and anti-PEG IgM produced significantly more C3a desArg and SC5b-9 in the presence of PLD, IL, L-PLD, and VL-PLD compared to control IgG and IgM antibodies (Figure 7). This indicates that even the very low levels of antibodies that bind to VL-PLD are sufficient to generate detectable complement activation. Under the conditions examined in this experiment, anti-PEG IgG generated similar levels of C3a *desArg* in the presence of PLD, IL, and L-PLD, but significantly less C3a desArg was generated in the presence of VL-PLD (Figure 7A). By contrast, significantly less SC5b-9 was generated by anti-PEG IgG for IL and L-PLD than for PLD (Figure 7B). No significant differences in either C3a *desArg* or SC5b-9 were measured when IL or L-PLD, which have the same density of PEG on their surface, were incubated with anti-PEG IgG. Less C3a *desArg* and SC5b-9 were generated in the presence of anti-PEG IgM as PEG levels decreased on liposomes (Figure 7C,D).

Hu6.3 anti-PEG IgG generated significantly greater C3a *desArg* (Figure S7A) and SC5b-9 (Figure S7B) than cAGP4 anti-PEG IgM for IL, L-PLD and VL-PLD. Anti-PEG IgG also generated more complement products than anti-PEG IgM for PLD, but the difference was not statistically significant. Interestingly, there appeared to be a threshold amount of both C3a desArg and SC5b-9 required for destabilization of liposomes; conditions producing complement product concentrations above the dashed line in Figure S7A,B correlated with liposome destabilization and high levels of drug release (Figure 6). By assuming: (1) all added anti-PEG antibodies are bound to surface DSPE-PEG molecules, (2) each DSPE-PEG molecule has one antibody-binding epitope, and (3) each anti-PEG IgG can occupy two PEG epitopes and each anti-PEG IgM can occupy 10 PEG epitopes, generation of C3a *desArg* (Figure 7E) and SC5b-9 (Figure 7E) significantly correlated to the theoretically estimated number of anti-PEG antibodies bound per liposome. These results support the idea that complement activation primarily correlates with the number of bound antibody molecules on liposomes rather than other liposome properties or differences in the encapsulated drug.

### Anti-PEG IgG-Mediated Liposome Destabilization Requires the Alternative Complement Pathway

**Figure 8:**
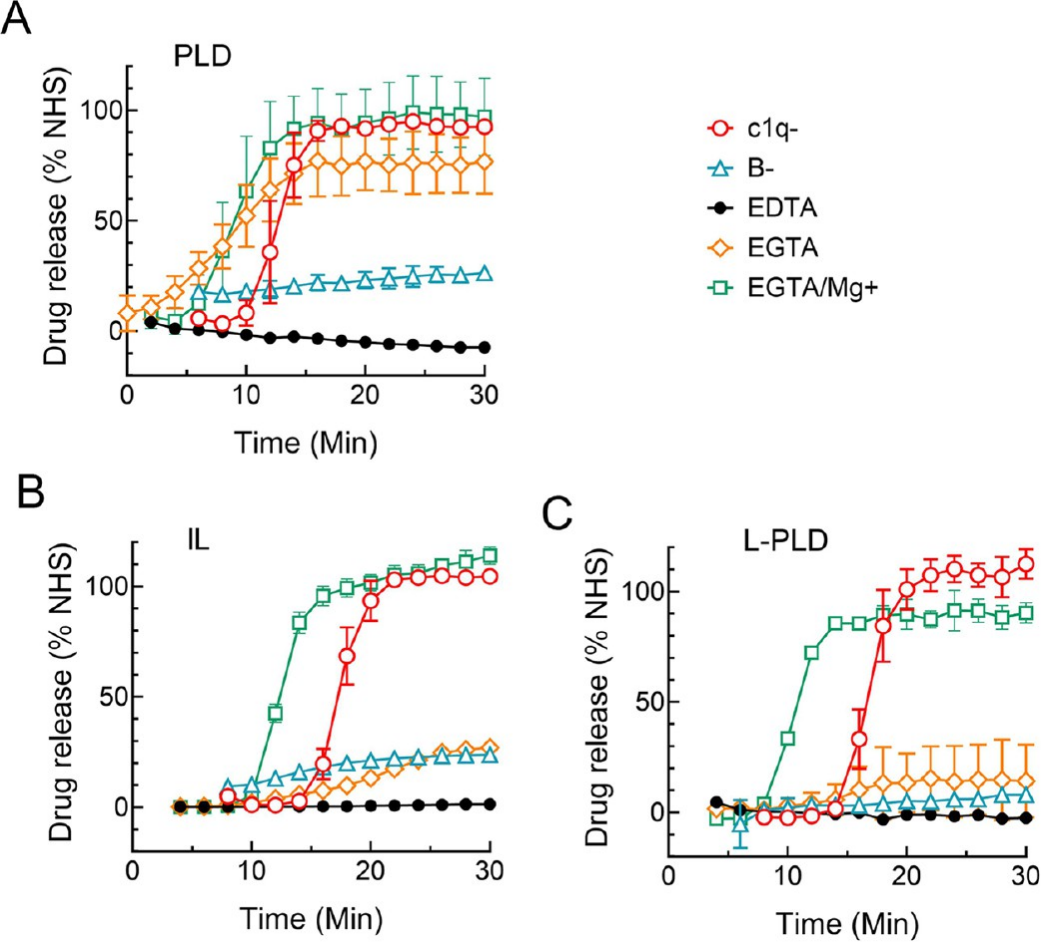
Complement pathways activated by human anti-PEG IgG. Time course of drug release from 68.5 μg mL^–1^ PLD (A), IL (B), or L-PLD (C) incubated with 150 μg mL^–1^ hu6.3 anti-PEG IgG. The release in the presence of chelating agents is normalized to release in NHS, release in C1q-deficient serum is normalized to release in the same serum supplemented with 100 μg mL^–1^ C1q, and release in factor *B* deficient serum is normalized to release in the same serum supplemented with 200 μg mL^–1^ factor *B*. Error bars show standard errors (*n* = 2).

The complement pathways responsible for anti-PEG induced drug release from the different liposomal formulations were examined. The effect of different treatments on the kinetics of drug release from each liposomal formulation was compared with drug release in normal human serum or depleted serum reconstituted with the depleted factor. Inhibition of all complement pathways by chelation of both Ca and Mg ions by ethylenediaminetetraacetic acid (EDTA) completely blocked drug release from PLD (Figure 8A), IL (Figure 8B), and L-PLD (Figure 8C), showing that complement is indeed required for liposome destabilization. Performing this experiment in serum deficient in C1q, which is required for initiation of the classical complement pathway, delayed release of drug from all liposomal formulations as compared to C1q-deficient serum reconstituted with C1q, but full release was eventually observed in all liposomes studied, showing that the classical pathway contributes but is not mandatory for liposome destabilization (Figure 8). By contrast, using serum deficient in Factor B, which is required for the alternative complement pathway, greatly reduced total drug release from all liposomes, indicating that the alternative complement pathway is primarily responsible for liposome destabilization. Only low levels of drug were released from IL and L-PLD in the presence of ethylene glycol-bis(β-aminoethyl ether)-*N*,*N*,*N*′,*N*′-tetraacetic acid (EGTA) which strongly chelates calcium ions to block the classical pathway and weakly binds Mg ions which hinders the alternative pathway, indicating that liposomal formulations having low levels of DSPE-PEG are particularly sensitive to impairment of the alternative pathway. Supplementation of EGTA with Mg (EGTA-Mg) to block the classical pathway but restore the full alternative pathway resulted in the delay of liposome destabilization; however, in agreement with the results of C1q-deficient serum, similar release as observed in normal human serum was eventually reached, confirming that the classical pathway can accelerate but is not required for drug release from liposomes.

### Cryo-EM Imaging of L-PLD

**Figure 9:**
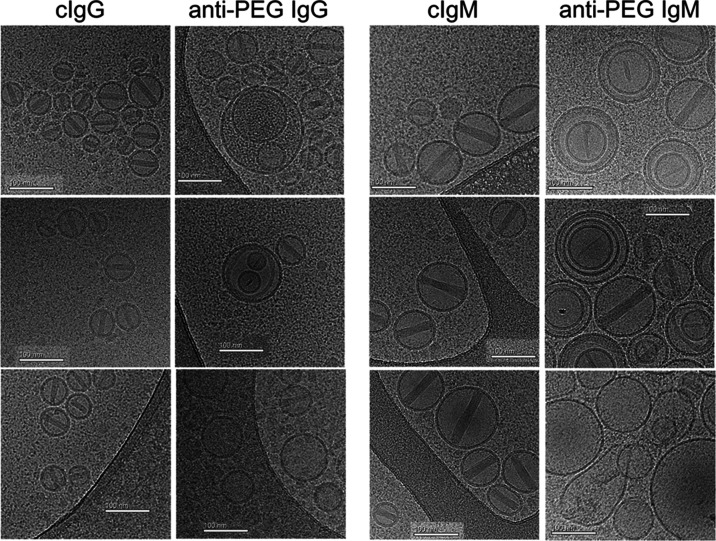
Cryo-TEM imaging of the effect of anti-PEG antibodies on L-PLD. L-PLD were incubated for 15 min at 37 °C in normal human serum containing 5 μg mL^–1^ human control IgG (cIgG), 5 μg mL^–1^ hu6.3 (anti-PEG IgG), 5 μg mL^–1^ human control IgM (cIgM), or 5 μg mL^–1^ cAGP4 human anti-PEG IgM (anti-PEG IgM). Scale bars show 100 nm.

To further understand how the PEG liposome coating affects drug release, we performed cryo-TEM imaging of L-PLD in the presence of anti-PEG antibodies. L-PLD displayed a similar morphology as PLD, with circular or slightly elliptical liposomes containing a single nanorod-like crystal of doxorubicin-sulfate in the intraliposome aqueous phase in the presence of a nonbinding control IgG antibody in normal human serum (Figures 9 and S8). However, anti-PEG IgG in normal human serum caused some L-PLD to form unilaminar and oligo-vesicular liposomes, sometimes with a small ammonium sulfate nanocrystal remaining in the lumen of the innermost liposomal layer (Figure 9). This morphology clearly differs from PLD, which remained uniformly unilaminar and either contained an intact doxorubicin-sulfate nanorod crystal or was completely empty (Figure 3 and ref (15)). Anti-PEG IgM also caused some L-PLD to form unilaminar and oligo-vesicular liposomes (Figures 9 and S9). Our results indicate that intraliposomal fusion is more prevalent at the lower PEG densities found on IL and L-PLD.

### Liposome Destabilization by Rat Anti-PEG IgG and IgM Antibodies

**Figure 10:**
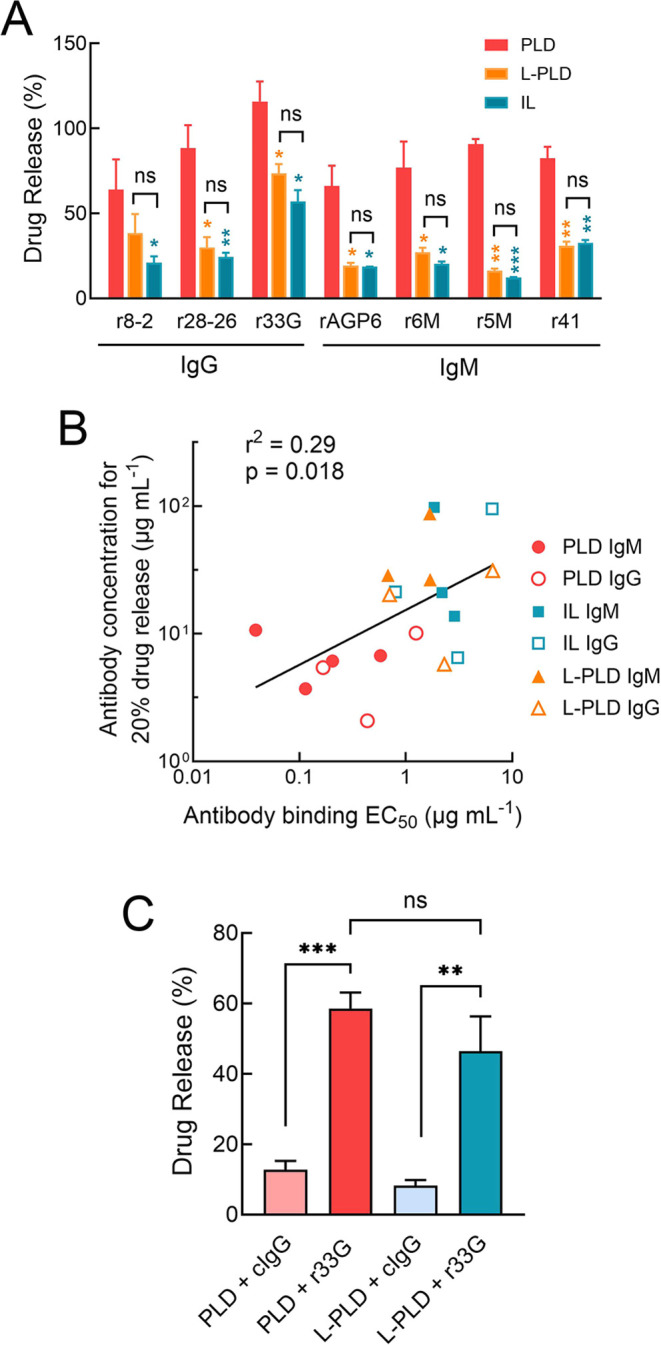
*In vitro* and *in vivo* liposome destabilization by rat anti-PEG antibodies. (A) Comparison of drug release from duplicate samples of PLD (red), L-PLD (orange), or IL (blue) in the presence of rat serum and 75 μg mL^–1^ of the indicated rat anti-PEG antibodies. Bars, SD. Significant differences in mean drug release from PLD and L-PLD or IL are indicated; *, *p* ≤ 0.05; **, *p* ≤ 0.01; ***, *p* ≤ 0.001. (B) Correlation between the antibody concentration required for 20% drug release from liposomes versus relative antibody binding avidity to liposomes. (C) Wistar rats were i.v. injected with 12.8 mg kg^–1^ control rat IgG or r33G rat anti-PEG IgG 30 min before i.v. injection of 5 mg kg^–1^ PLD or L-PLD. Doxorubicin release from liposomes was measured in plasma 5 min later. Results show the mean values from three rats. Bars, SD. Significant differences in mean values: ns, not significant; **, *p* ≤ 0.01; ***, *p* ≤ 0.005.

We generated a panel of three rat IgG monoclonal antibodies and four rat IgM monoclonal antibodies that bind to PEG to further understand how anti-PEG antibodies affect liposome stability. In common with human anti-PEG antibodies, more IgG and IgM rat anti-PEG antibodies bound to PLD as compared to IL and L-PLD, reflecting the higher density of PEG on the surface of PLD (Figure S10). The relative binding avidities of the antibodies were estimated by calculating the EC_50_ values (50% response) on sigmoidal dose–response curves fit to the ELISA data (Table 2). rAGP6 displayed the highest binding to PLD whereas r28-26 bound most strongly to both IL and L-PLD. Incubation of the liposomes in the presence of normal rat serum and the anti-PEG antibodies but not control IgG or IgM resulted in liposome destabilization in a dose-dependent manner (Figure S11). In general, both anti-PEG IgG and IgM induced greater drug release from PLD as compared to L-PLD and IL. Comparison of the drug release induced by a fixed concentration (75 μg mL^–1^) of the antibodies showed that there was no significant difference in the drug release from IL and L-PLD, but release from PLD was significantly higher for all the antibodies other than r8-2 (Figure 10A). This indicates that as found for anti-PEG IgG and IgM, drug release *in vitro* is largely determined by the PEG density and the number of antibodies bound to the liposomes. There was also a weak correlation between drug release and antibody binding avidity, suggesting that antibodies that bind PEG on liposomes more strongly can induce greater drug release (Figure 10B).

**Table 2:** Binding Avidities of Rat Anti-PEG Antibodies

		binding EC_50_ (μg mL^–1^)		
antibody	class	PLD	IL	L-PLD
r8-2	IgG	1.3	6.5	6.6
r33G	IgG	0.44	3.1	2.3
r28-26	IgG	0.17	0.80	0.70
rAGP6	IgM	0.039	1.9	1.7
r5M	IgM	0.21	6.0	10
r6M	IgM	0.11	2.8	0.7
r41-9	IgM	0.58	2.2	1.7

We previously demonstrated that anti-PEG IgG can destabilize PLD integrity in rats.^15^ Here we determined if anti-PEG antibodies could also destabilize liposomes with lower PEG densities. We injected Wistar rats with a rat anti-PEG IgG monoclonal antibody (r33G), waited 30 min for the antibody to distribute throughout the rats, and then injected PLD or L-PLD. We chose to use L-PLD instead of IL because we previously validated a published method to separate doxorubicin present in liposomes from free doxorubicin in plasma samples.^15,29,30^ The dose of rat IgG used in this experiment is high but within the upper range of anti-PEG IgG concentrations found in humans.^4,5^ About 60% of doxorubicin was released from PLD in rats injected with r33G as compared to about 45% for L-PLD, although the mean release was not significantly different (Figure 10C). Since we measured both total serum doxorubicin (total intact PLD plus released doxorubicin) and also doxorubicin only present in intact liposomes, the percent drug release offers a snapshot of liposome stability at the time of sample collection. These results confirm that *in vivo* in rats anti-PEG antibodies can destabilize liposomes with both high (5%) and low (0.3%) levels of PEG modification.

Previous studies have demonstrated that PEGylated nanomedicines such as PEGylated liposomal doxorubicin (PLD) can be destabilized by binding of anti-PEG antibodies to PEG on the nanoparticle surface followed by complement activation that results in liposome damage.^15,16^ Here we investigated if the clinically used nanomedicine irinotecan liposomal (IL), which includes only 0.3 mol % DSPE-PEG in its membrane, can evade destabilization by anti-PEG antibodies. By contrast, PLD incorporates about 16-fold higher levels of DSPE-PEG (5.3 mol %). We found that more anti-PEG antibodies bound to PLD, resulting in greater complement activation and drug release as compared to IL. Complement activation and liposome destabilization correlated with the theoretically estimated number of IgG or IgM antibodies bound to the surface of the liposomes. The mechanisms of drug release from IL and PLD are distinct; PLD destabilized by anti-PEG IgG retained their basic liposome size but displayed discrete membrane defects, presumable due to the insertion of membrane attack complexes in the liposome membrane. On the other hand, the release of irinotecan from IL induced by anti-PEG IgG depended on liposome fusion. Anti-PEG IgM was able to induce the formation of large unilaminar and oligo-vesicular liposomes from both PLD and IL. We also generated L-PLD with 0.3 mol % DSPE-PEG (similar to IL) and VL-PLD with 0.01 mol % DSPE-PEG. Drug release levels, mechanism of drug release, complement activation levels, and complement pathways were similar for IL and L-PLD, indicating that the DSPE-PEG levels and not the encapsulated drug is the major determinant of anti-PEG antibody action on liposomes. VL-PLD also bound low levels of anti-PEG antibodies that induced complement activation, but the level of activated complement was insufficient to destabilize the liposomal formulation.

IL has an average diameter of about 110 nm, while PLD is about 90 nm in diameter. Doxorubicin is remotely loaded into PLD by the creation of a transmembrane ammonium gradient concomitant with high intraliposome sulfate concentrations which act as a counteranion to form a single nanorod crystal of doxorubicin-sulfate per liposome that is approximately 70 nm long and 20 nm wide, resulting in loading of approximately 10,000 doxorubicin molecules per liposome.^21,31−33^ Irinotecan, which like doxorubicin is an amphipathic weak base, is remotely loaded into liposomes by the creation of a transmembrane triethylammonium (TEA) gradient and high intraliposome concentrations of sucrose octasulfate, which acts as a counteranion to irinotecan to form a stable irinotecan gel/precipitate, resulting in loading of over 100,000 irinotecan molecules per liposome.^17^ Both liposomal drugs have methoxy PEG_2000_ on their lipid surface, but IL incorporates about 16-fold less PEG than PLD (0.3 mol % DSPE-PEG versus 5.3 mol %, respectively). Assuming equal molar ratios of DSPE-PEG to total lipid molecules in the inner and outer leaflets of the liposomes, we estimate that there are approximately 1900, 160 and 110, and 4 PEG_2000_ molecules on the surface of PLD, IL, L-PLD, and VL-PLD, respectively (Table 3). The estimated surface densities of PEG on IL and L-PLD are identical. At the low density on IL and L-PLD, PEG forms a “mushroom” conformation in which each PEG chain moves independently whereas on PLD, PEG chains contact each other, forming a “brush” configuration that stabilizes liposomes by combined dehydration of the lipid headgroup region along with increased hydration of the outer PEG layer.^34−36^

**Table 3:** Comparison of PEG on Liposomes^a^,^c^

liposome	diameter (nm)	PEG (mol %)	surface PEG molecules	PEG surface density (PEG/nm^2^)	*R*_F_/*D*^b^	PEG form
PLD	90	5.3	1900	0.075	2.4	brush
IL	110	0.3	160	0.0043	0.06	mushroom
L-PLD	90	0.3	110	0.0043	0.06	mushroom
VL-PLD	90	0.01	4	0.00016	0.002	mushroom

a The estimated number of PEG molecules on the surface of each liposome, PEG surface density, and structural form of PEG on the liposomes are shown.

b Flory radius of PEG *R*_F_ = *aN*^3/5^, where *a* = ethylene oxide monomer length (0.35 nm) and *N* = number of ethylene oxide repeats (45.5) for PEG_2000_. Grafting distance *D* = 2(*A*/π)^1/2^ where *A* = area occupied per PEG chain which corresponds to 1/ρ, where ρ = PEG density.^42^

c PEG forms a mushroom conformation when *R*_F_/*D* ≤ 1 and a brush conformation when *R*_F_/*D* > 1.^43^

PEG in the brush conformation can improve liposome stability, increase liposome circulation time, and enhance tumor penetration.^37,38^ However, high grafting densities of PEG can hinder liposome interactions with other cells due to the association of a large number of water molecules with each PEG chain, resulting in a hydration shell surrounding the liposome surface.^35,39^ Endocytosis of PLD may not be required to achieve antitumor activity because the accumulation of ammonia in the tumor microenvironment due to the cancer cell metabolic pathway of glutaminolysis, which continuously generates ammonia that can directly induce the release of doxorubicin from intact liposomes.^40^ Although irinotecan is also an amphipathic weak base and may be released by ammonia in the tumor environment, irinotecan is a prodrug, which requires the action of carboxylesterases to form the active drug SN-38. In addition, the lower density of PEG on the surface of IL allows the formation of a prominent protein corona of serum proteins on the liposome surface (visible in Figure 3B,D), which is less apparent on the highly PEGylated PLD. This protein corona may promote cell interactions and uptake of IL into cancer cells and tumor-infiltrating macrophages for effective anticancer activity.^17,41^

We observed some general trends for both human and rat anti-PEG antibody interactions with PEGylated liposomes. First, there was a positive correlation between the theoretically estimated number of anti-PEG antibodies bound to each liposome and the degree of complement activation. Thus, the lower density of PEG on IL and L-PLD resulted in significantly less anti-PEG antibody binding and complement activation as compared to PLD. Surprisingly, very low but significant levels of both C3a *desArg* and SC5b-9 were generated even when VL-PLD were incubated with anti-PEG antibodies, demonstrating that binding of just a few anti-PEG antibodies to liposomes can activate the complement cascade. Non-PEGylated liposomes can activate complement but neither we in our current study nor Szebeni and colleagues^44^ observed significant complement activation for Doxil, possibly because small, electrically neutral liposomes are not very potent complement activators and we examined lower liposomes concentrations than many previous studies.^45−48^

Strong drug release from liposomes was observed when about 20–30 hu6.3 anti-PEG IgG or cAGP4 anti-PEG IgM antibodies bound per liposome. We estimate that there are only four PEG chains present on VL-PLD, preventing the binding of sufficient numbers of antibodies to induce strong drug release. These results indicate that reduction of PEG levels on lipid nanoparticles may not be a realistic method to overcome antibody-mediated destabilization since such a low PEG density would be unlikely to provide the beneficial effects of PEG such as particle stabilization and prolongation of *in vivo* half-life. On the positive side, drug release was not observed from liposomes in the presence of either human or rat control antibodies in serum. Natural immunoglobulins present in serum can bind to nanoparticles, including PLD, and activate the complement cascade.^49^ Our *in vitro* results indicate that under the conditions used in our study neither control IgG or IgM nor immunoglobulins present in normal human or rat serum bound to PLD, IL, or L-PLD in sufficient numbers to cause liposome destabilization and drug release.

Another trend observed in our study was that liposome destabilization was positively correlated with relative rat anti-PEG antibody binding avidity. This held for both IgG and IgM antibodies and probably relates to more antibody accumulation on the liposomes as the antibody binding avidity increases. This result suggests that lipid nanoparticles may be relatively resistant to destabilization by pre-existing anti-PEG antibodies present in a large percentage of the population as these antibodies are believed to possess low binding affinity to PEG.^7^

Anti-PEG IgM is often considered to induce more robust complement activation and hypersensitivity reactions than anti-PEG IgG,^16,50,51^ but anti-PEG IgG induced more complement activation and drug release from PEGylated liposomes in our study. We used rat or humanized antibodies in our study, which may activate complement differently than the porcine and rabbit anti-PEG antibodies used in previous studies. Human anti-PEG IgG also accelerates clearance of PEGylated proteins and liposomes at least as well as anti-PEG IgM.^14,52,53^ We further found that anti-PEG IgG but not anti-PEG IgM induces hypersensitivity reactions against PEGylated liposomes, nanoparticles and proteins in mice.^26^ In our experience, strong biological effects can be initiated by anti-PEG IgG antibodies.

The elongated shape of Doxil along with free doxorubicin nanocrystals contribute to stronger complement activation as compared to drug-free liposomes.^44^ However, L-PLD and VL-PLD do not take into account the effect that the particle shape may have on complement activation. Our results using control nonbinding IgG or IgM antibodies indicate that complement activation is much stronger in the presence of anti-PEG antibodies than in their absence. We previously showed that the binding of anti-PEG IgG and IgM to spherical, empty DOXEBO liposomes was identical to Doxil, indicating that the particle shape and the presence of loaded drug or drug nanorod crystals do not affect the binding of anti-PEG antibodies.^54^ Our finding that there is almost no difference in the *in vitro* behavior of Onyvide and L-PLD to anti-PEG antibodies and that they both differ from Doxil strongly suggests that the mole% of DSPE-PEG is the main factor in the response to anti-PEG antibodies.

Complement activation by anti-PEG IgG largely depended on the alternative complement pathway, regardless of the PEG density on the liposomes. Inhibition of the classical complement pathway, which requires physical binding of at least two (and optimally six) adjacent IgG molecules for effective C1q binding and activation,^55,56^ delayed but did not prevent liposome destabilization by anti-PEG IgG. By contrast, inhibition of the alternative complement pathway, which provides a continuous source of activated C3, completely blocked drug release from PLD, IL, and L-PLD induced by anti-PEG IgG. PLD induced stronger activation of the alternative pathway as compared to IL and L-PLD as shown by less sensitivity to partial inhibition of the alternative pathway by EGTA and by a reduced delay in drug release when the classical pathway was inhibited. The alternative complement pathway was therefore strictly required for liposome destabilization by anti-PEG IgG whereas the classical pathway accelerated drug release from PLD, IL, and L-PLD. These results are consistent with accumulating evidence that the alternative pathway is the predominant pathway induced by antibodies bound to the surface of nanoparticles and liposomes with the classical pathway providing a secondary role.^15,16,49^ Vu and colleagues proposed a seed hypothesis model in which a few molecules of C3b are first deposited on nanoparticle-bound IgG molecules, which is known to protect bound C3b from inactivation by the soluble complement regulators factors H and I.^49,57^ These C3b molecules can act as seed sites for cleavage by factor *D* to form C3bBb and properdin-stabilized C3 convertases which amplify the alternative pathway by enzymatically generating large amounts of C3b.^58^ Taken together, our results indicate that PEG density affects the degree, but not the mechanisms, of complement activation by anti-PEG antibodies bound to liposomal formulations.

**Figure 11:**
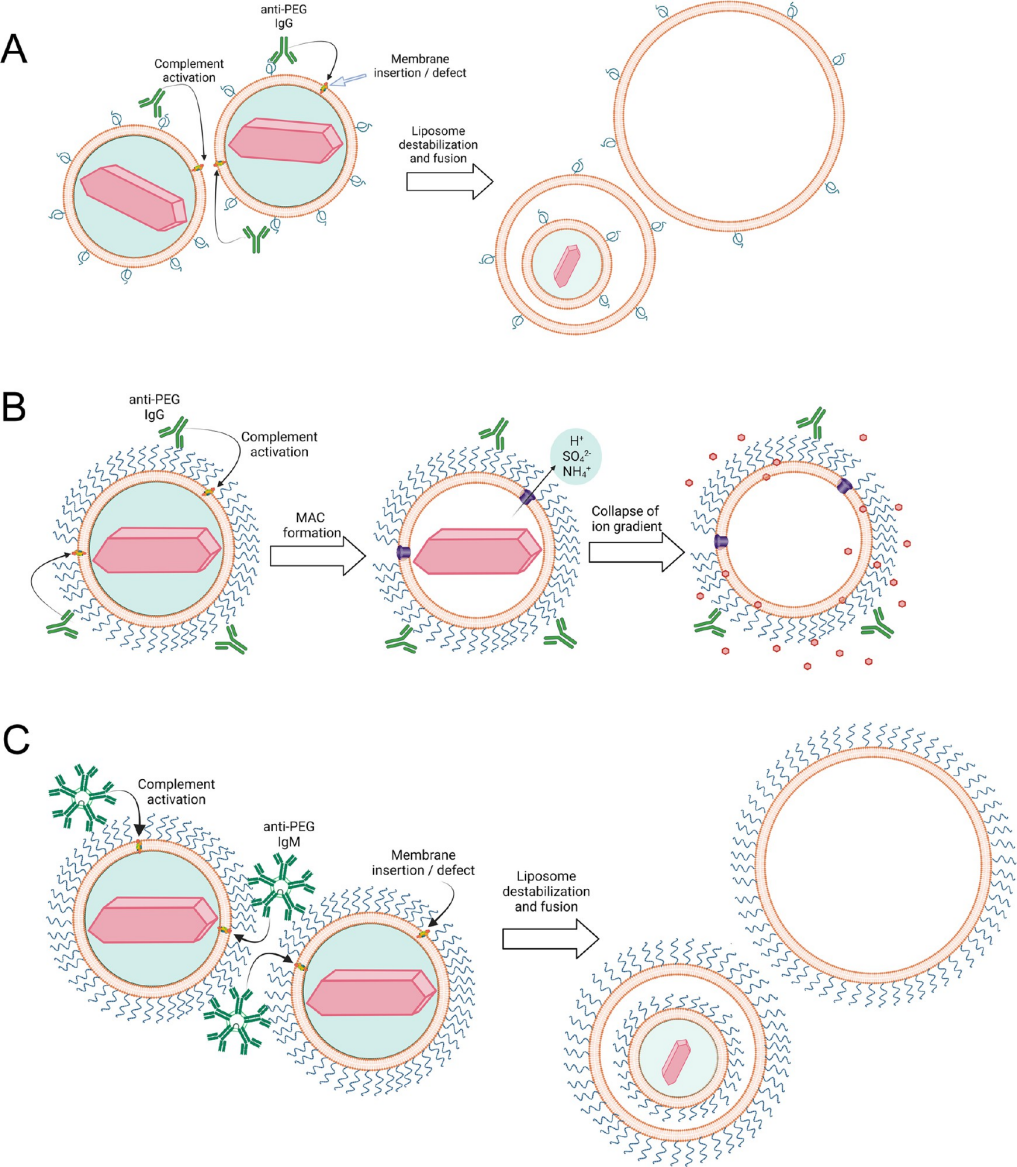
Proposed mechanism of liposome destabilization by anti-PEG antibodies. (A) Anti-PEG IgG or IgM bound to L-PLD can activate the complement cascade, resulting in the deposition of terminal complement proteins in the lipid membrane, resulting in membrane defects, which cause intraliposomal fusion to form large multilaminar and multivesicular liposomes. (B) The dense layer of PEG present on PLD hinders intraliposomal fusion caused by anti-PEG IgG complement activation, allowing the formation of the membrane attach complex in individual liposomes. The loss of the ion gradient in the liposomes allows rapid diffusion of doxorubicin from affected liposomes. (C) The multivalent nature of anti-PEG IgM can overcome the PEG barrier on PLD to promote intraliposomal fusion and the formation of large multilaminar and multivesicular liposomes.

The mechanism of liposome destabilization depended on the density of PEG on liposomes, as well as the structure of the anti-PEG antibody. Destabilization of IL and L-PLD by anti-PEG IgG seemed to proceed by fusion of liposomes to form large liposomes as well as multilamellar and multivesicular liposomes (Figures 3C and 9). The terminal product of the complement cascade (the membrane attack complex, C5b-9) is a barrel-like protein complex that forms a large ∼10 μm channel in lipid membranes.^59^ Formation of the membrane attack complex depends on insertion and conformational changes of β-pore forming proteins that destabilize and cause rupture of lipid bilayers.^60^ We hypothesize that alignment of defect-rich domains near anti-PEG antibodies exposes hydrophobic membrane domains and therefore in accordance with the “hydrophobic effect” leads to liposome aggregation and membrane fusion, resulting in the formation of large multilaminar or multivesicular liposomes (Figure 11A).^61,62^ Bilayer fusion occurs within a millisecond,^62^ consistent with the retention of small doxorubicin-sulfate nanocrystals in the innermost liposome of some multivesicular vesicles, indicating that fusion was sufficiently rapid to maintain a partial transmembrane ammonium ion gradient. In contrast to IL and L-PLD, the C5b-9 channel can be visualized in PLD by cryo-TEM, resulting in destabilized PLD that maintains their original size but lack the doxorubicin-sulfate nanocrystal (Figure 3C and ref (15)). The channel disrupts the ammonium and pH gradients in PLD, allowing rapid dissolution of the intraliposomal doxorubicin-sulfate nanorod crystals and drug diffusion out of PLD^31,33^ (Figure 11B). We suggest that the dense layer of PEG on PLD hinders liposome aggregation and fusion, allowing maturation of the membrane attack complex in individual liposomes.^63,64^ Anti-PEG IgM induced the formation of multilamellar and multivesicular PLD, suggesting that the multivalent nature of IgM may be able to promote close contact between aggregated PLD to overcome the PEG barrier and allow PLD fusion (Figure 11C).

Although our *in vitro* studies clearly demonstrate that anti-PEG antibodies can activate complement and destabilize liposomes with only 0.3% PEG, we wanted to test whether liposomes displaying low levels of PEG can be destabilized by anti-PEG antibodies *in vivo*. The binding of anti-PEG antibodies to liposomes accelerates their clearance from the blood, primarily by enhanced uptake into resident macrophages in the liver.^7,53,65,66^ It is, therefore, possible that liposomes are cleared from the circulation before they are destabilized. However, we found that a rat anti-PEG IgG antibody (r33G) induced significant complement activation and drug release (∼50%) from both PLD and L-PLD in rats. We used a relatively high concentration of r33G in this experiment (∼250 μg mL^–1^) rather than a lower concentration that might be representative of the anti-PEG antibody levels found in many people because liposomes are safe and stable in the vast majority of patients.^67^ Rather, it is the few patients who have high levels of anti-PEG antibodies that are of concern. We previously measured anti-PEG IgG levels of up to 238 μg mL^–1^ in normal individuals without prior exposure to PEGylated medicines or CoV-2 lipid nanoparticle mRNA vaccines, which incorporate PEG-lipids in their formulations.^7^ SARS-CoV-2 lipid nanoparticle mRNA vaccines increase the concentrations of anti-PEG IgG and IgM in the circulation of vaccine recipients.^68−70^ High concentrations of anti-PEG antibodies are also induced in some patients exposed to immunogenic PEGylated medicines.^8,71−74^ For example, patients treated with pegloticase, a PEGylated form of porcine uricase, can develop anti-PEG titers exceeding 1,000,000, which is likely a higher concentration of anti-PEG antibodies than we examined.^72^ The prevalence and concentrations of anti-PEG antibodies may increase as lipid nanoparticle vaccines and therapeutics become more widespread, which in turn might lead to problems in liposome or lipid nanoparticle stability in patients with high concentrations of anti-PEG antibodies in their circulation.

## Conclusions

Although IL is grafted with low densities of PEG, this liposomal formulation remains susceptible to drug release caused by anti-PEG antibodies. Anti-PEG IgG antibodies activate complement to create a pore on PLD whereas liposomes with lower PEG densities (IL and L-PLD) are destabilized by the fusion of liposomes to create large multivesicular liposomes. Anti-PEG IgM can activate complement and promote the formation of multivesicular and multilaminar liposomes from PLD and IL. Complement activation and liposome destabilization correlate with the theoretically estimated number of antibodies bound per liposome, but even liposomes with very low levels of PEG can activate the complement cascade. Our results suggest that reducing the levels of DSPE-PEG is not a viable solution to prevent liposome destabilization by anti-PEG antibodies.

## Methods/Experimental Details

### Materials

ELISA kits from Quidel (San Diego, CA) were used to detect human SC5b-9 (A020) and human C3a (A031). Rat complement C3 ELISA kit (ab157731) was purchased from Abcam (Waltham, Boston). Normal human serum (A113), human sera depleted of C1q (A509), and human sera depleted of factor *B* (A056), C1q protein (A400), and factor *B* protein (A408) were also from Quidel. Male Wistar rats (11 weeks old, 250–300 g) were from BioLASCO Taiwan (Taipei, Taiwan). Animals were housed under standard light/dark cycles and had access to food and water ad libitum. Animal experiments were conducted according to institutional guidelines and ethically approved by the Laboratory Animal Facility of the Institute of Biomedical Sciences, Academia Sinica.

### Antibodies

Humanized anti-PEG IgG (hu6.3), humanized antimethoxy PEG IgG (hu15-2b), and chimeric human anti-PEG IgM (cAGP4) antibodies were developed in our lab and have been previously described.^4,15^ Humanized means that all antibody sequences are human, whereas chimeric human IgM means that the constant regions of the antibody are derived from human IgM but the variable regions are the original murine sequences. We refer to these as human anti-PEG antibodies, for simplicity. Control human IgG and IgM antibodies were obtained from Sigma-Aldrich (St. Louis, MO). Rat monoclonal antibodies were generated by immunizing female Sprague–Dawley rats as previously described for mouse monoclonal antibodies.^19^ Three rat IgG antibodies (r33G, r28-26, and r8.2) and four rat IgM (rAGP6, r5M, r6M, and r41) were generated. Control rat IgG (p12) and rat IgM (Xten) were also generated in our lab as described.^15^ Anti-PEG antibodies used in our study are summarized in Table S1.

### Liposomal Drugs

PLD (LC101), an FDA-approved generic Doxil, was kindly provided by Ayana Pharma (Jerusalem, Israel). LC101 has a diameter of ∼90 nm with a doxorubicin concentration of 2 mg mL^–1^ and lipid content of 16 mg mL^–1^ is composed of hydrogenated soy phosphatidylcholine (HSPC): cholesterol: *N*-(carbonyl-methoxypoly(ethylene glycol)-2000)-1,2-distearoly *sn*-glycero-3-phosphoethanolamine (DSPE-PEG_2000_) in a molar ratio of 56.6:38.1:5.3.^15^ The HSPC is a mixture of distearoylphosphatidylcholine mixed with 1-palmitoyl-2-stearoylphosphatidylcholine in a mole ratio ranging from 72/28 to 66/34. The *T*_m_ is in the range of 53–51 °C reflecting the ratio of acyl chains.^20^ PLD with 0.3 mol % (L-PLD) or trace (0.01 mol %) PEG (VL-PLD) were generated by reducing the amount of DSPE-PEG_2000_ and increasing the amount of HSPC. All PLDs used in this study are suspended in 10% sucrose in 10 mM histidine buffer, pH 6.5. Doxorubicin, an amphipathic weak base, was remotely/actively loaded into the nanoliposomes at >95% efficiency driven by a transmembrane ammonium gradient. Loading was stabilized by the high concentration of interliposome sulfate counteranions (250 mM) that resulted in the formation intraliposome doxorubicin-sulfate nanorod crystals^21^

IL (Onivyde), kindly provided by Kaohsiung Medical University, has an average size of ∼110 nm and is composed of 1,2-distearoyl-*sn*-glycero-3-phosphocholine (DSPC), cholesterol, and DSPE-PEG_2000_ in a molar ratio of 59.8, 39.9 and 0.3.^22,23^ Thus, IL has 1 molecule of DSPE-PEG per 333 lipid molecules as compared with 1 per 20 molecules in Doxil. Other excipients of IL include sucrose-octasulfate, 2-[4-(2 hydroxyethyl)piperazin-1-yl] ethanesulfonic acid (HEPES buffer), sodium chloride, and water for injection. Irinotecan is an amphipathic weak base that is remotely/actively loaded into the intraliposome aqueous phase with >95% efficiency due to a triethylammonium transmembrane gradient. Loading is stabilized by the high intralumen concentration of sucrose-octasulfate, which is the counteranion of the triethylammonium, that forms a gel/precipitate with irinotecan in the intraliposome aqueous phase.^22^ The compositions of PLD, L-PLD, VL-PLD, and IL are summarized in Table S2.

### PEG-Binding ELISA

Antibody binding to liposomes was performed as described with minor modifications.^15^ EIA microplates were coated with 0.25 μg per well of m24G (mouse antimethoxy PEG IgG). Plates were washed once with PBS and then blocked with 5% skim milk powder in PBS. PLD, L-PLD, VL-PLD, and IL were diluted to 20 μg mL^–1^ (total lipid concentration) in 2% skim milk/PBS, then serially diluted 5-fold in 2% skim milk/PBS before dilutions were added to the ELISA plate. After 60 min at room temperature, excess liposomes were removed by washing the wells twice with PBS before hu6.3, hu15-2b, or cAGP4 antibodies (2.5 μg mL^–1^; 50 μL per well) were added for 1 h. The wells were washed three times with PBS before HRP-conjugated streptavidin (0.5 μg/well) was added for 1 h. After washing plates three times with PBS, peroxidase activity was quantified by the addition of 2,2′-azino-bis(3-ethylbenzothiazoline-6-sulfonic acid) diammonium salt (ABTS) and H_2_O_2_ (3000:1) for 30 min before reading the absorbance at 405 nm.

### Complement ELISA

Hu6.3, cAGP4, or control human IgG and IgM antibodies were diluted to 300 μg mL^–1^ in GHBS^2+^ buffer (0.1% gelatin, 5 mM HEPES, 145 mM NaCl, 0.15 mM CaCl_2_, and 0.5 mM MgCl_2_, pH 7.3). PLD and IL were diluted to 137 or 68.5 μg mL^–1^ of total lipids in normal human serum. Equal 50 μL volumes of the liposome and antibody solutions in triplicate were mixed together for 15 min at 37 °C. The samples were immediately placed on ice, and then the concentrations of the complement products C3a *desArg* or SC5b-9 were measured by ELISA following the manufacturer’s instructions (Quidel, San Diego, CA). For experiments that included V-PLD and VL-PLD, the concentrations of antibodies were reduced to 150 μg mL^–1^ to give a final antibody concentration of 75 μg mL^–1^.

### Drug Release from Liposomes

Drug release from liposomes was performed as previously described.^15^ Briefly, anti-PEG antibodies were first diluted to 300 μg mL^–1^, then serially diluted 2-fold in GHBS^2+^ buffer. Human serum containing PLD, L-PLD, VL-PLD, or IL (137 or 68.5 μg mL^–1^ total lipid concentration) were added to the antibody dilutions at a 1:1 ratio, so that the highest antibody concentration was 150 μg mL^–1^ and the total lipid concentration was 68.5 or 34.5 μg mL^–1^ (as indicated in figure legends). Mixtures were incubated at 37 °C for 30 min in a black microtiter plate. Drug release was determined by measuring the fluorescence on a microplate reader (TECAN Infinite M1000 Pro). Fluorescence of mixtures containing PLD was read at 490/590 nm (excitation/emission, respectively), while mixtures containing IL were read at 375/400 nm (excitation/emission). 100 and 0% drug release were estimated by replacing antibody solutions with an equal volume of 15% Triton X-100 or GHBS^2+^ buffer, respectively. Percentage of drug release is calculated as (fluorescence reading – 0% reading)/(100% reading – 0% reading) × 100.

### Complement Pathways

IL, L-PLD or PLD (34.25 μg mL^–1^ of lipid) and 150 μg mL^–1^ Hu6.3 anti-PEG IgG in GHBS^2+^ buffer plus 50% complement-deficient human serum or GHBS^0^ buffer (0.1% gelatin, 5 mM HEPES, 145 mM NaCl, pH 7.3) plus 50% human serum for chelating agent experiments were incubated at 37 °C in black microtiter plates. Fluorescence readings were taken every 2 min as described above to measure drug release. EDTA or EGTA was added to a final concentration of 10 mM in human sera and kept at room temperature for at least 30 min to chelate calcium and magnesium ions. The addition of 20 mM MgCl_2_ into EGTA-treated sera restored the alternative pathway while still inhibiting the classical pathway. Drug release from each liposome was compared to drug release from the same liposome in NHS for chelating agents or to C1q-deficient serum and Factor B deficient serum that was supplemented with 100 μg mL^–1^ of C1q or 400 μg mL^–1^ of factor B to restore classical or alternative complement pathways, respectively.

### Cryogenic Electron Microscopy

PLD, IL, or L-PLD were incubated anti-PEG antibodies and their isotype control antibodies in human serum or PBS for 15 min at 37 °C. After resuspending thoroughly, 4 μL of each sample was pipetted onto 200 nm mesh holey carbon grids (Electron Microscopy Sciences) and blotted for 3 s. Grids were observed on a FEI Tecnai F20 operating at 200 kV and images acquired at 50,000-fold magnification (2528 e nm^–2^).

### *In Vivo* Drug Release

A similar procedure as previously reported was used to examine drug release from PLD and L-PLD *in vivo*.^15^ Male Wistar rats were i.v. injected with 12.8 mg kg^–1^ r33G rat anti-PEG IgG or control rat IgG 30 min before the rats were i.v. injected with mg kg^–1^ of PLD or L-PLD. Rats were anesthetized by isoflurane inhalation, and blood was withdrawn by cardiac puncture into K_2_-EDTA tubes within 5 min of receiving liposomes. Samples were immediately centrifuged for 12 min at 1500*g* at 4 °C and plasma was transferred into a clean polypropylene tube. Plasma samples were diluted 4-fold with PBS and aliquoted into separate tubes for drug release or complement assays.

Released doxorubicin in plasma samples was measured as described.^15^ Briefly, the cation exchanger Dowex-50-hydrogen was converted to the sodium form by successive washes with 2 M NaOH, 1 M NaCl, and 0.9% NaCl. Dowex-50 strongly binds doxorubicin in solution but does not bind doxorubicin encapsulated in liposomes. 600 μL plasma aliquots were either untreated (total intact PLD or L-PLD plus released doxorubicin) or treated with 100 mg of Dowex to remove free doxorubicin (leaving just intact PLD). The samples were gently mixed for 20 min, centrifuged at 1500*g* for 1 min, and then duplicate 100 μL samples of each plasma sample were transferred to a black fluorescence microtiter plate into wells containing 100 μL PBS. Doxorubicin fluorescence was measured on a Tecan Infinite M1000 Pro at 490/590 nm. The percentage of free doxorubicin (DOX) in comparison to total doxorubicin (free DOX plus PLD DOX) was estimated from fluorescence measurements of untreated (*F*) and Dowex-treated (FD) plasma samples. The difference in *F* – FD represents the fluorescence of free doxorubicin in plasma samples.

### Statistical Analysis and Calculations

All graphs and statistical analyses were performed using Graphpad Prism 7.0 (La Jolla, CA). Significance between treatment groups was analyzed by the unpaired Student’s *t* test. The number of lipid molecules present in the outer (*N*_outer_) and inner (*N*_inner_) leaflets of the liposomes was calculated by *N*_outer_ = 17.69 (*d*/2)^2^ and *N*_inner_ = 17.69 (*d*/2–5)^2^ where *d* is the liposome diameter in nm.^24^ Because X-ray diffraction shows that DSPE-PEG in the inner and outer membrane leaflets has similar electron densities,^25^ we assume an equal mole percent of DSPE-PEG in the two leaflets.

## Supporting Information Available

Characteristics of anti-PEG antibodies; comparison of pegylated liposomal drugs used in this study; control IgG and IgM do not induce drug release from PLD and IL; cryo-TEM of PLD; cryo-TEM of IL; anti-PEG antibody disruption of IL depends on active complement; cryo-TEM examining the effect of anti-PEG IgM on PLD; cryo-TEM examining the effect of anti-PEG IgM on IL; complement activation and drug release by anti-PEG IgG and IgM antibodies; cryo-TEM examining the effect of anti-PEG IgG on L-PLD; cryo-TEM examining the effect of anti-PEG IgM on L-PLD; rat anti-PEG antibody binding to liposomes; and liposome destabilization by rat anti-PEG antibodies (PDF)

## Abbreviations

DSPE-PEG: 1,2-distearoyl-*sn*-glycero-3-phosphoethanolamine-poly(ethylene glycol)
IgG: immunoglobulin G
IgM: immunoglobulin M
IL: irinotecan liposomes
PLD: PEGylated liposomal doxorubicin
L-PLD: PEGylated liposomal doxorubicin with 0.3 mol % DSPE-PEG
PEG: poly(ethylene glycol)
VL-PLD: PEGylated liposomal doxorubicin with 0.01 mol % DSPE-PEG
