# Supplementary material for: Liposomes with Low Levels of Grafted Poly(ethylene glycol) Remain Susceptible to Destabilization by Anti-Poly(ethylene glycol) Antibodies
Source: ACS Nano. 2024 Aug 9;18(33):22122–38. doi: 10.1021/acsnano.4c05409 (PMC11342370; doi:10.1021/acsnano.4c05409)
Supplement: Supplementary file 1 — nn4c05409_si_001.pdf [file nn4c05409_si_001.pdf]

SUPPORTING INFORMATION

Liposomes with Low Levels of Grafted Polyethylene Glycol Remain Susceptible to Destabilization by Anti-Polyethylene Glycol Antibodies

Bing-Mae Chen[1], Even Chen[1], Yi-Chen Lin[1, 2], Trieu Thi My Tran[1], Keren Turjeman[3], Shih-Hung Yang[1], Tian-Lu Cheng[4], Yechezkel Barenholz[3]*, and Steve R Roffler[1,4]*

[1]Institute of Biomedical Sciences, Academia Sinica, Taipei 11529, Taiwan.

[2]Graduate Institute of Life Sciences, National Defense Medical Center, Taipei 11490, Taiwan.

[3]Department of Biochemistry and Molecular Biology, Hebrew University-Hadassah Medical School, Jerusalem 91120, Israel.

[4]Graduate Institute of Medicine, College of Medicine, Kaohsiung Medical University, Kaohsiung 80708, Taiwan.

*Correspondence to:

Dr. Steve R. Roffler, Room N233, Institute of Biomedical Sciences, Academia Sinica, No. 128, Section 2, Academia Road, Taipei 11529, Taiwan. Tel: (886)-2-2652-3079; Email: sroff@ibms.sinica.edu.tw

Dr. Yechezekel Barenholz, Laboratory of Membrane and Liposome Research, The Hebrew University-Hadassah Medical School, IMRIC, Jerusalem 91120, Israel. Email: chezyb1@gmail.com

Supplemental Table 1: Characteristics of anti-PEG antibodies

**Antibody**	**Species**	**Class**	**Specificity**
hu6.3	Human	IgG_1	PEG backbone
hu15-2b	Human	IgG_1	methoxyPEG
cAGP4	Chimeric human	IgM	PEG backbone
r8-2	Rat	IgG_{2c}	PEG backbone
r28-26	Rat	IgG_{2a}	PEG backbone
r33G	Rat	IgG_{2a}	PEG backbone
rAGP6	Rat	IgM	PEG backbone
r6M	Rat	IgM	PEG backbone
r5M	Rat	IgM	PEG backbone
r41	Rat	IgM	PEG backbone

Supplemental Table 2: Comparison of pegylated liposomal drugs used in this study

**Formulation**	PLD (Pegylated liposomal doxorubicin)	IL (Onivyde, Irinotecan liposomal)	L-PLD (Low PEG liposomal doxorubicin)	VL-PLD (Very low PEG liposomal doxorubicin)
**Description**	The first FDA-approved liposomal drug. Used for treating multiple cancers	Minimally pegylated liposomal irinotecan for the treatment of pancreatic cancer	PLD with a similar mole percentage of PEG as IL	PLD without PEG
**Diameter**	90 nm	110 nm	90	90
**Drug concentration**	2 mg mL^{-1}	5 mg mL^{-1}	2 mg mL^{-1}	2 mg mL^{-1}
**Lipid type**	HSPC	DSPC	HSPC	HSPC
**Lipid (mole %)**	56.6%	59.8%	59.8%	60.1%
**Cholesterol (mole %)**	38.1%	39.9%	39.9%	39.9%
**PEG-Lipid (mole %)**	5.3%	0.3%	0.3%	0.01%

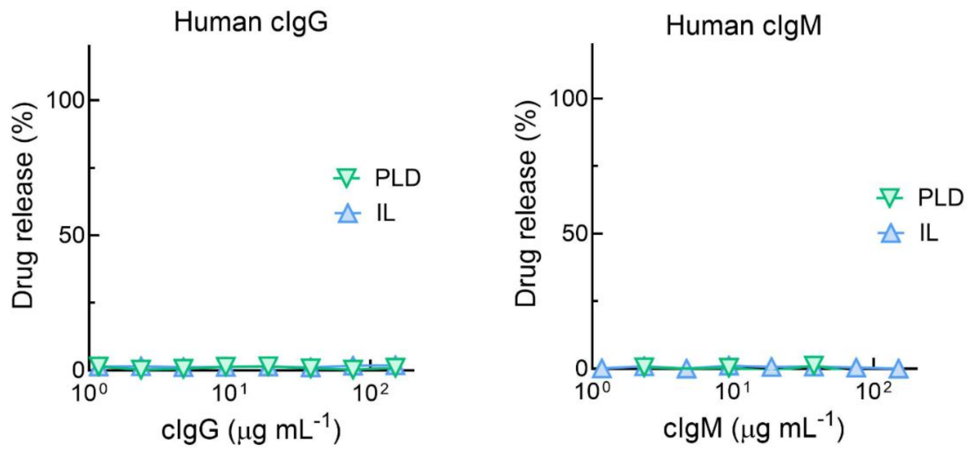

Supplemental Figure 1. Control IgG and IgM do not induce drug release from PLD and IL. Drug release was measured from 34 µg mL^{-1} (based on lipid) PLD or IL incubated with the indicated concentrations of non-binding human IgG (**cIgG**) or human IgM (**cIgM**) for 30 minutes at 37°C in human sera. (*n* = 3).

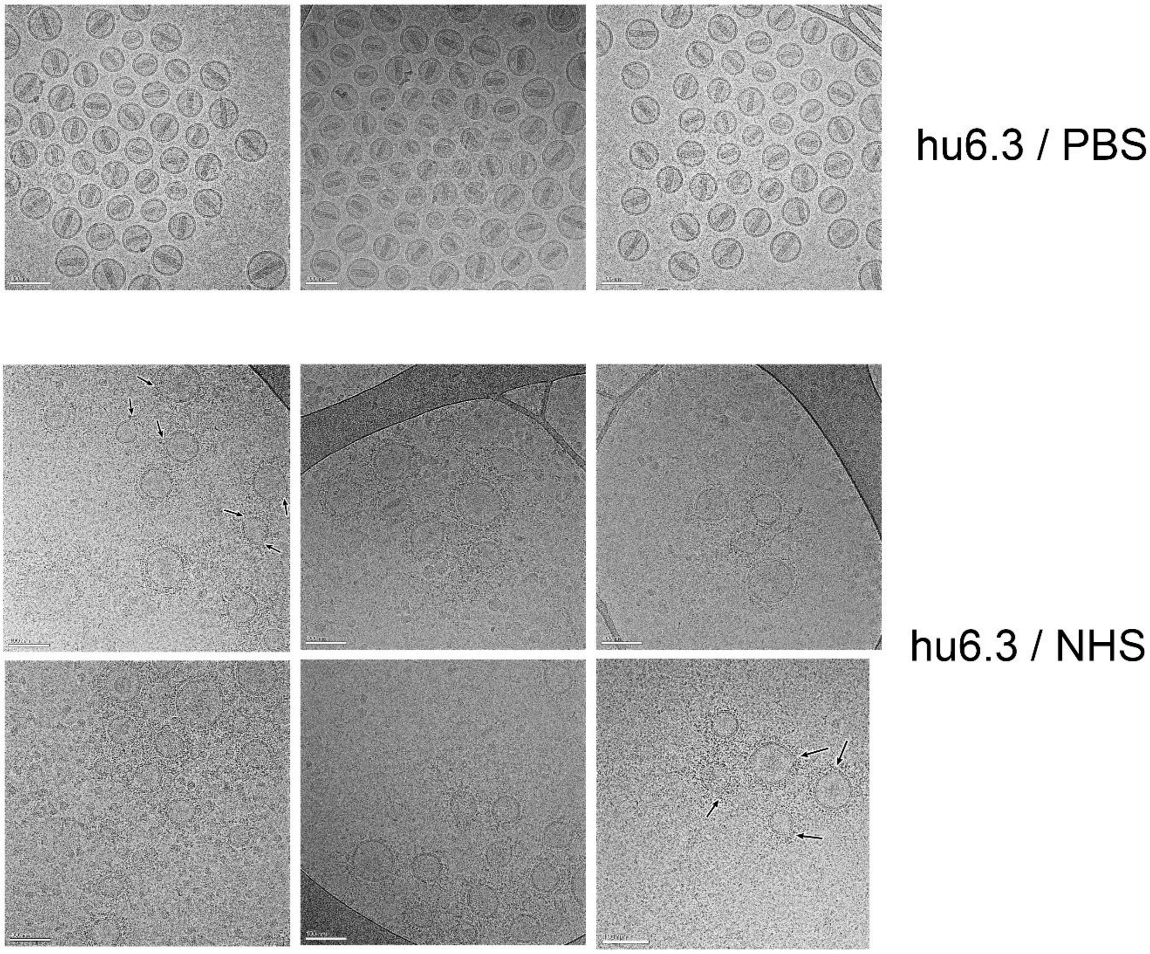

Supplemental Figure 2. Cryo-TEM of PLD. PLD were incubated for 15 minutes at 37°C in PBS containing 5 µg mL^{-1} hu6.3 anti-PEG IgG **(hu6.3 / PBS**) or normal human serum containing 5 µg mL^{-1} hu6.3 anti-PEG IgG (**hu6.3 / NHS**). Black arrows indicate areas where the membrane has been damaged by complement. All scale bars show 100 nm.

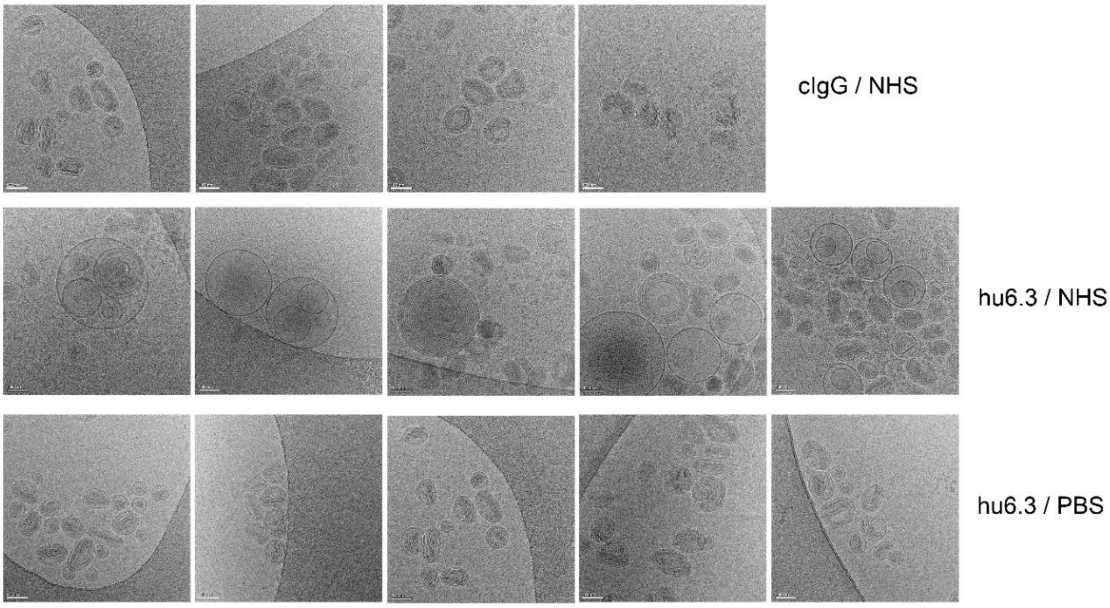

Supplemental Figure 3. Cryo-TEM of IL. IL were incubated for 15 minutes at 37°C in normal human serum containing 5 µg mL^{-1}control human IgG_1 **(cIgG / NHS**), normal human serum containing 5 µg mL^{-1} hu6.3 anti-PEG IgG (**hu6.3 / NHS**), or PBS containing 5 µg mL^{-1} hu6.3 anti-PEG IgG (**hu6.3 / PBS**). All scale bars show 100 nm.

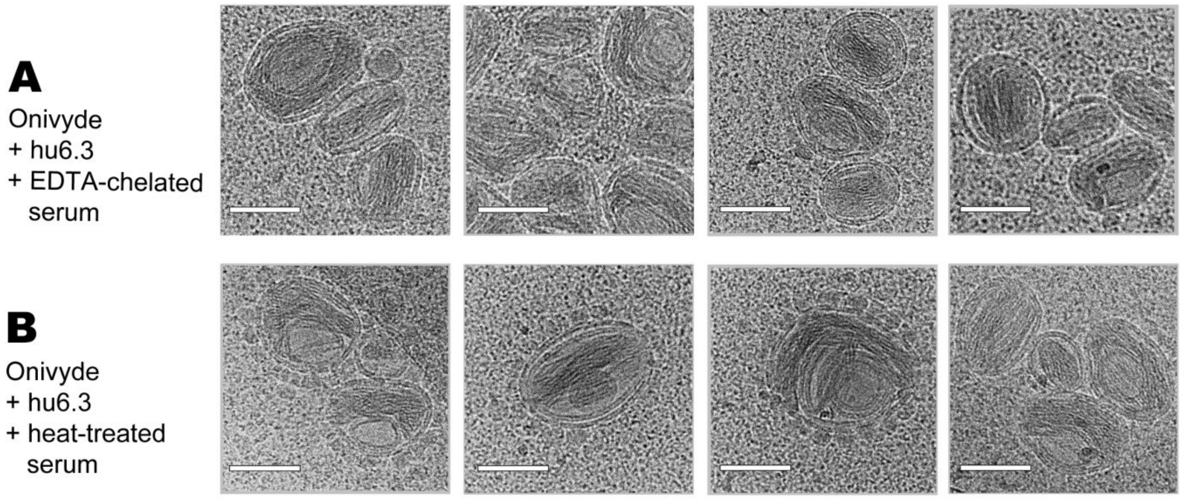

Supplemental Figure 4. Anti-PEG antibody disruption of IL depends on active complement. Serum was treated by **A)** Ion chelation by EDTA, and **B)** heated to 56 degrees to compromise complement activity. Incubation with hu6.3 and IL shows severely impaired drug lysis. Scale bars show 100 nm.

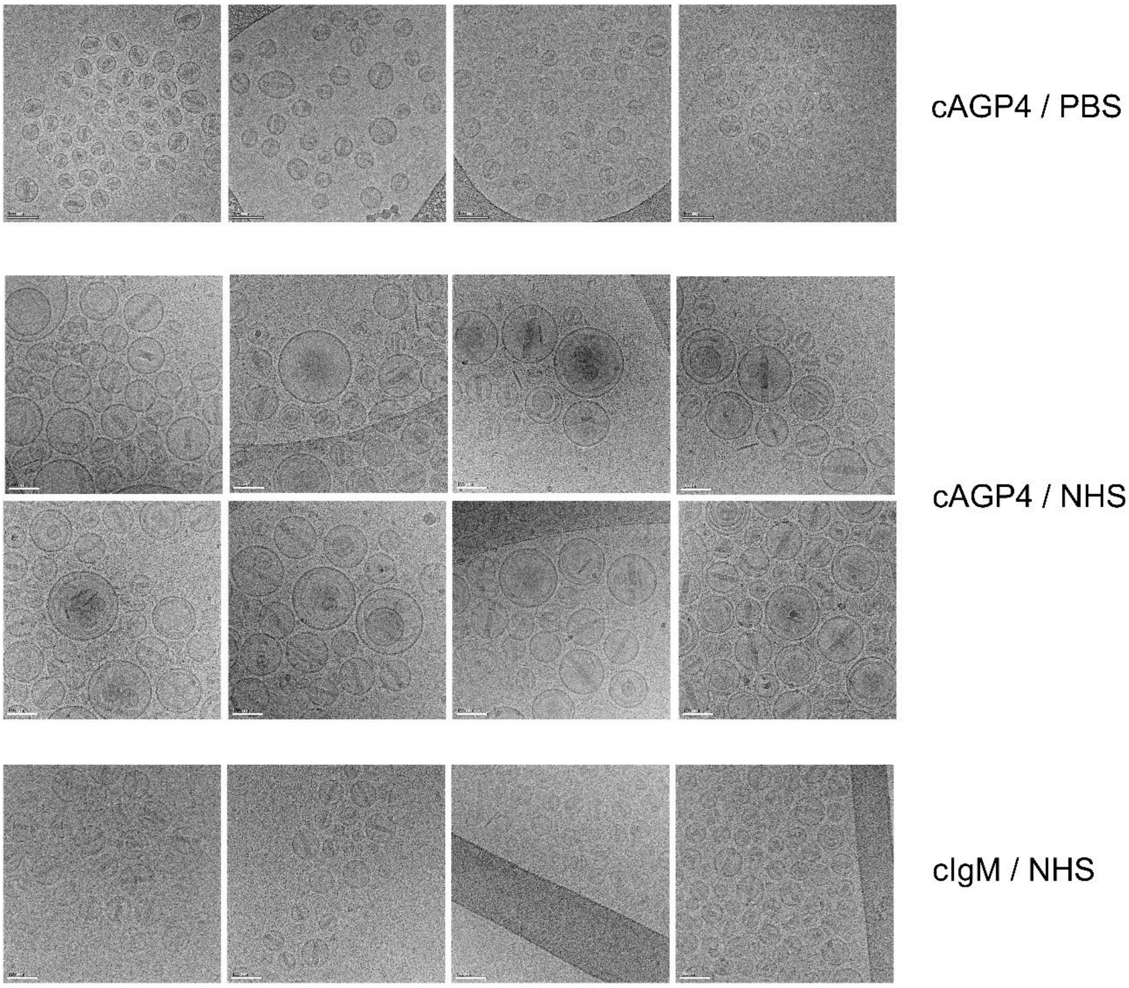

Supplemental Figure 5. Cryo-TEM examining the effect of anti-PEG IgM on PLD. PLD were incubated for 15 minutes at 37°C in PBS containing 5 μg mL^{-1} cAGP4 anti-PEG IgM (**cAGP4 / PBS**), normal human serum containing 5 μg mL^{-1} cAGP4 anti-PEG IgM (**cAGP4 / NHS**), or normal human serum containing 5 μg mL^{-1} control human IgM (**cIgM / NHS**). Scale bars = 100 nm.

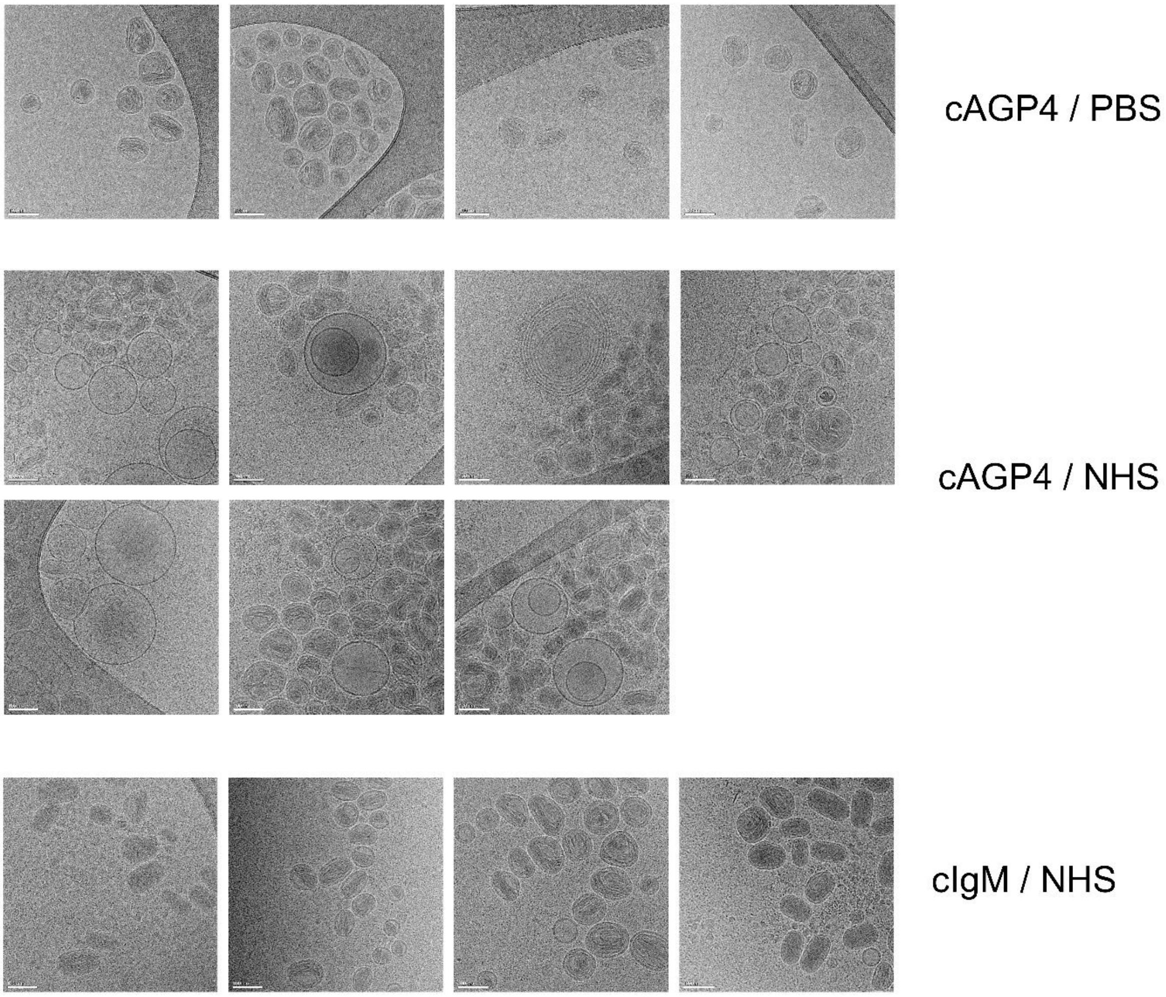
cAGP4 / PBS
cAGP4 / NHS
cIgM / NHS

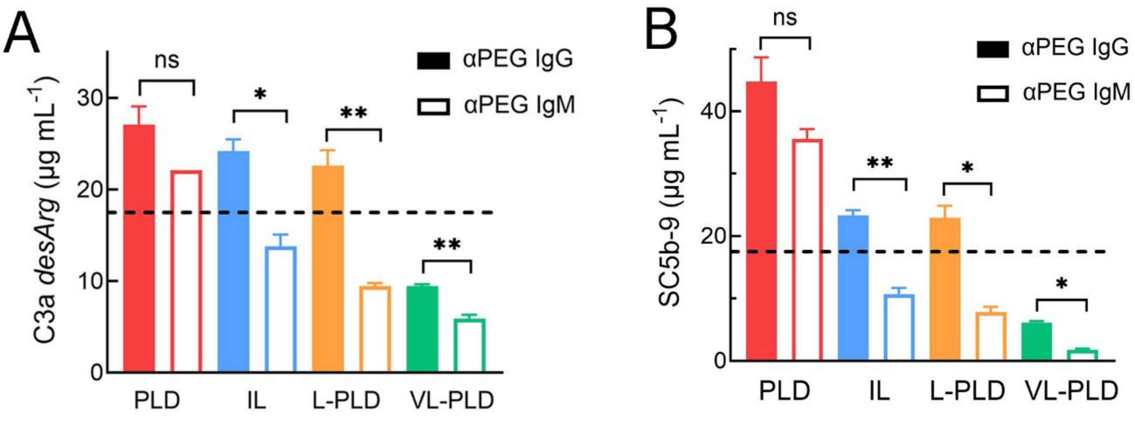

Supplemental Figure 7. Complement activation and drug release by anti-PEG IgG and IgM antibodies. Mean concentrations of C3a *desArg* (**A**) and SC5b-9 (**B**) after incubation of 68.5 µg mL^{-1} PLD, IL, L-PLD, or VL-PLD with anti-PEG IgG or IgM antibodies in normal human serum for 15 minutes. Bars, SD. Statistical significance between complement reaction products generated in the presence of anti-PEG IgG versus IgM are indicated: ns, not significant; *, $p \leq 0.05$; **, $p \leq 0.005$. The dashed lines indicate the approximate threshold for liposome destabilization with complement product values above the dashed line corresponding to strong drug release.

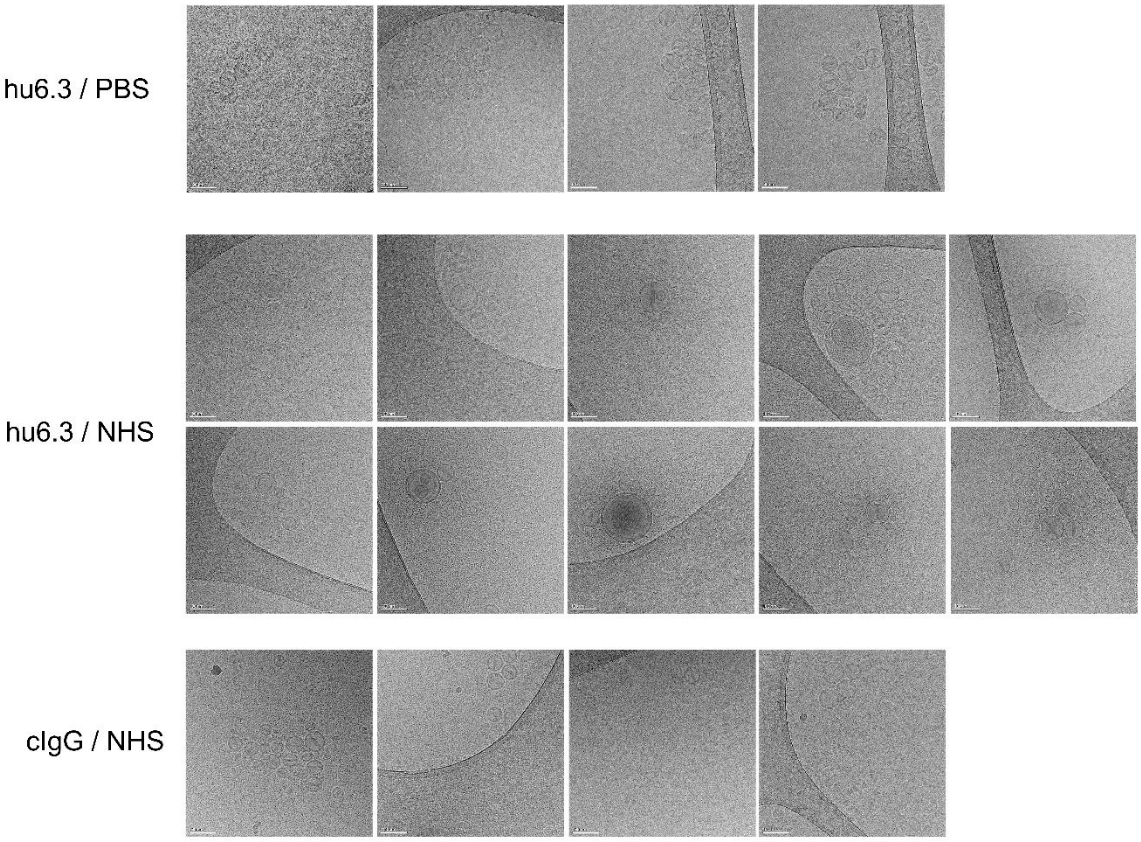

Supplemental Figure 8. Cryo-TEM examining the effect of anti-PEG IgG on L-PLD. L-PLD were incubated for 15 minutes at 37°C in PBS containing 5 μg mL^{-1} hu6.3 anti-PEG IgG (**hu6.3 / PBS**), normal human serum containing 5 μg mL^{-1} hu6.3 anti-PEG IgG (**hu6.3 / NHS**), or normal human serum containing 5 μg mL^{-1} control human IgG (**cIgG / NHS**). Scale bars = 100 nm.

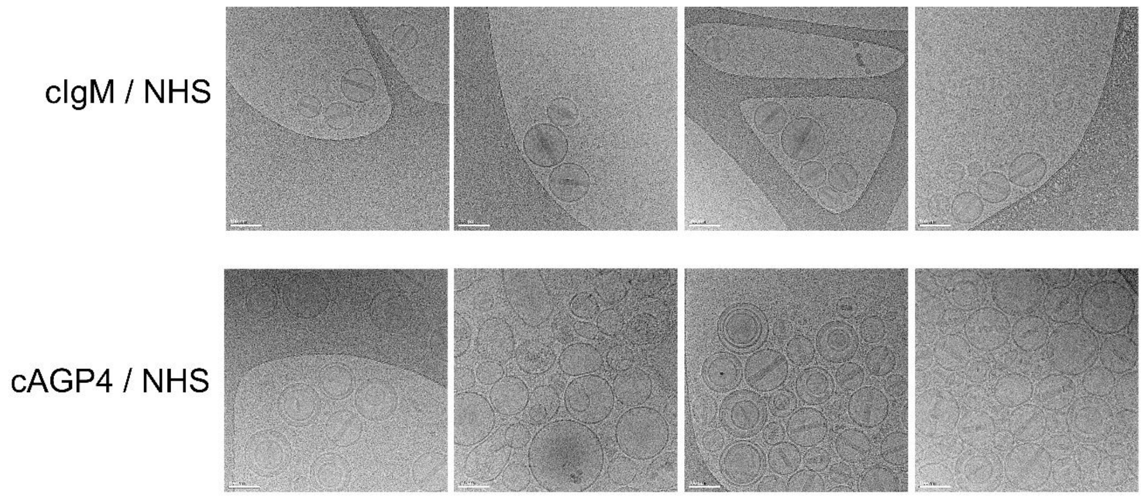

Supplemental Figure 9. Cryo-TEM examining the effect of anti-PEG IgM on L-PLD. L-PLD were incubated for 15 minutes at 37°C in normal human serum containing 5 µg mL^{-1} control human IgM (**cIgM + NHS**) or normal human serum containing 5 µg mL^{-1} cAGP4 anti-PEG IgM (**cAGP4 + NHS**). Scale bars = 100 nm.

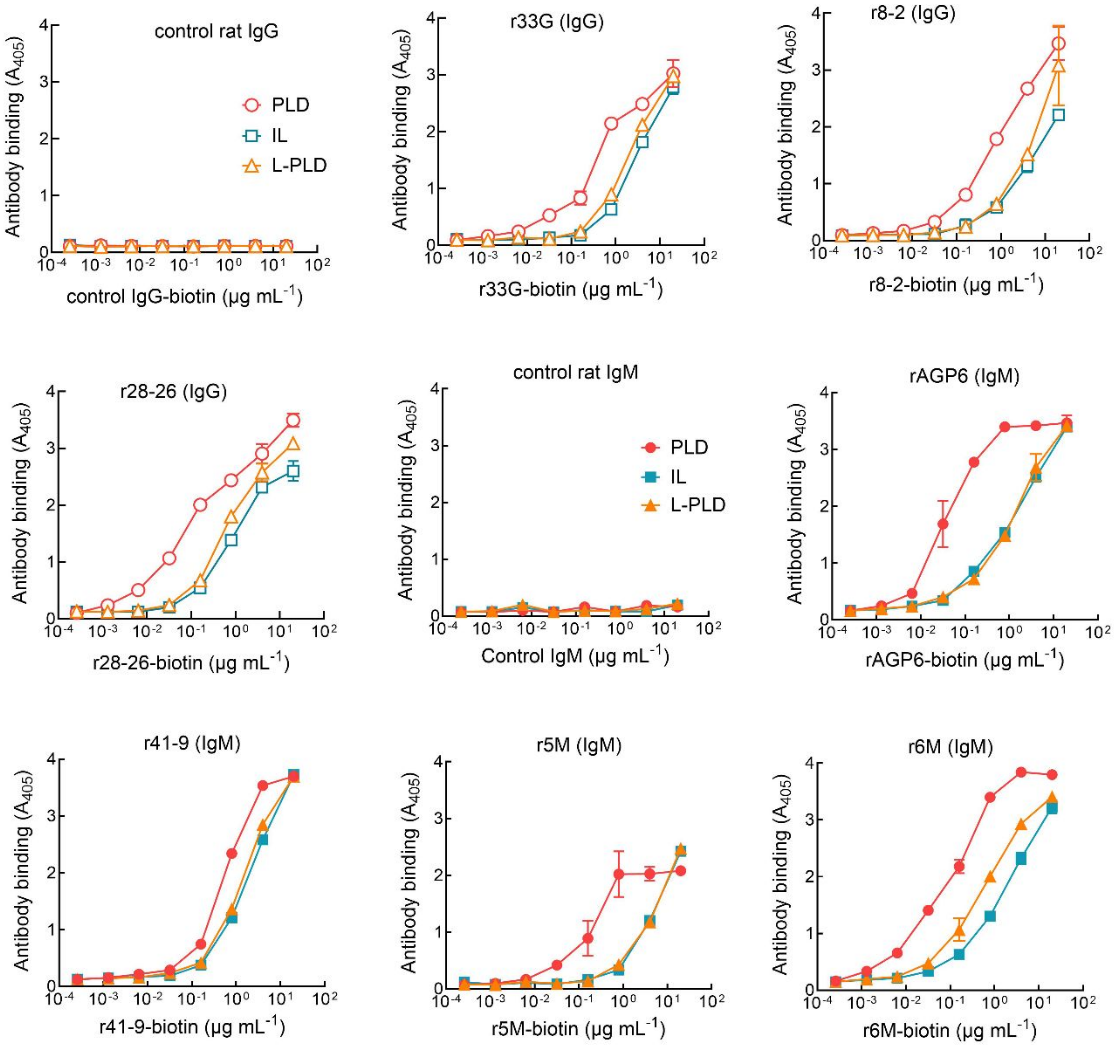

Supplemental Figure 10. Rat anti-PEG antibody binding to liposomes. Binding of 2.5 μg mL^{-1} rat biotin-labelled anti-PEG IgG or IgM antibodies to the indicated concentrations (based on lipids) of PLD, IL or L-PLD in duplicate are shown. cIgG and cIgM are negative control rat antibodies. Bars show mean values and standard deviations.

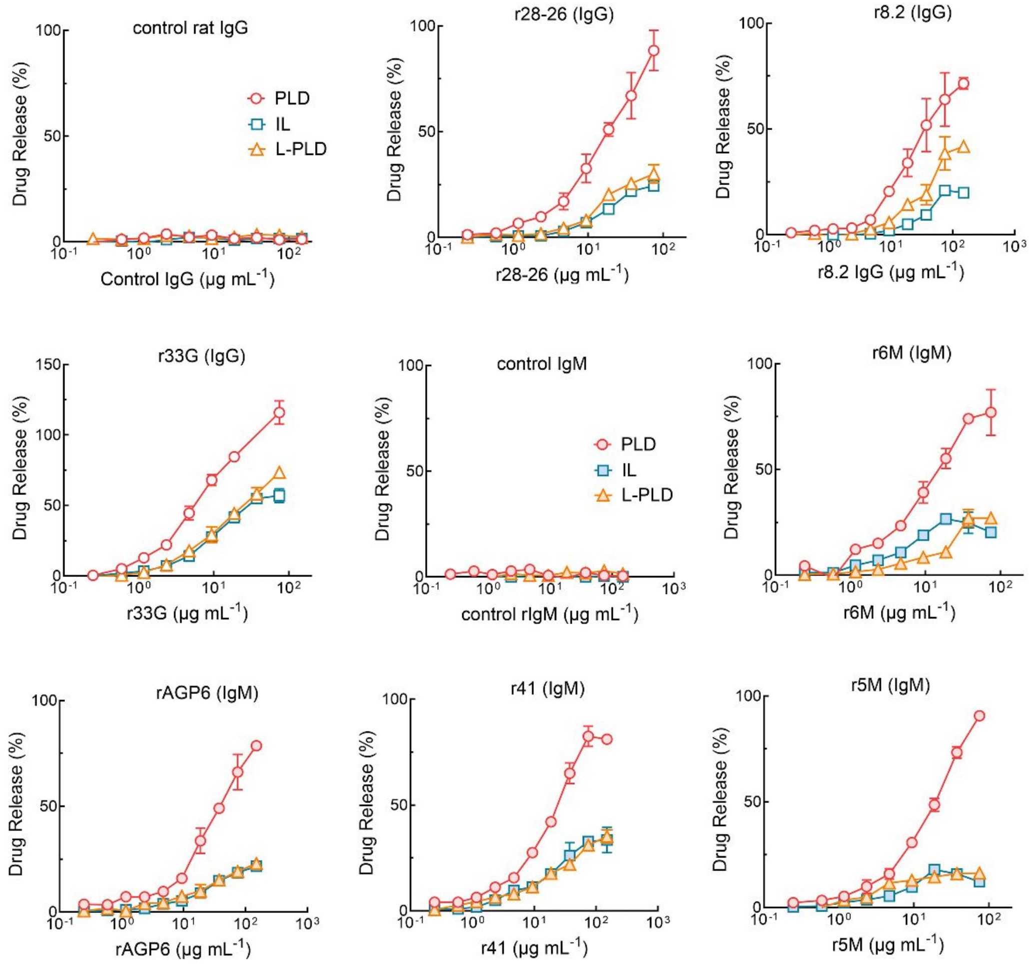

Supplemental Figure 11. Liposome destabilization by rat anti-PEG antibodies. Drug release was measured from duplicate samples of PLD, IL, or L-PLD in the presence of normal rat serum and the indicated concentrations of rat control or anti-PEG antibodies. Bars, SEM.
